# Multiple functions of the crustacean gill: osmotic/ionic regulation, acid-base balance, ammonia excretion, and bioaccumulation of toxic metals

**DOI:** 10.3389/fphys.2012.00431

**Published:** 2012-11-15

**Authors:** Raymond P. Henry, Čedomil Lucu, Horst Onken, Dirk Weihrauch

**Affiliations:** ^1^Department of Biological Sciences, Auburn UniversityAuburn, AL, USA; ^2^Center for Marine Research Rovinj, Institute Ruđder Bošković ZagrebRovinj, Croatia; ^3^Department of Aquaculture, University of DubrovnikDubrovnik, Croatia; ^4^Department of Biological Sciences, Wagner CollegeStaten Island, NY, USA; ^5^Department of Biological Sciences, University of ManitobaWinnipeg, MB, Canada

**Keywords:** crustaceans, gills, osmoregulation, ion transport, ammonia excretion, toxicology, acid-base balance

## Abstract

The crustacean gill is a multi-functional organ, and it is the site of a number of physiological processes, including ion transport, which is the basis for hemolymph osmoregulation; acid-base balance; and ammonia excretion. The gill is also the site by which many toxic metals are taken up by aquatic crustaceans, and thus it plays an important role in the toxicology of these species. This review provides a comprehensive overview of the ecology, physiology, biochemistry, and molecular biology of the mechanisms of osmotic and ionic regulation performed by the gill. The current concepts of the mechanisms of ion transport, the structural, biochemical, and molecular bases of systemic physiology, and the history of their development are discussed. The relationship between branchial ion transport and hemolymph acid-base regulation is also treated. In addition, the mechanisms of ammonia transport and excretion across the gill are discussed. And finally, the toxicology of heavy metal accumulation via the gill is reviewed in detail.

## Introduction and background

The study of osmoregulation in crustaceans goes back over 100 years, during which both major technological and conceptual advances have been made and fundamental core concepts have been reaffirmed. The crustacean gill, which functions in osmoregulation, has also been shown to play a central role in multiple other physiological and biochemical processes. The estuarine environment, which is harsh to begin with because of variations in naturally occurring physical parameters, is made more difficult because it is also typical the site of accumulation of anthropogenically produced toxic materials, such as heavy metals. This purpose of this review is to treat the topic of multiple gill functions comprehensively, from the ecological perspective to the level of genome. In doing so, our goal is to tie together the classical concepts of the foundational literature with the recent advances in physiology, molecular, and genomic biology, uniting a vast body of literature in one resource. Also, in addition to treating the functions of the gill in a reductionist, mechanistic framework, this review also attempts to integrate these mechanisms into the complex functions of the gill within the organism, and the organism within the environment.

### The environmental challenges and benefits of euryhalinity

**Figure 1:**
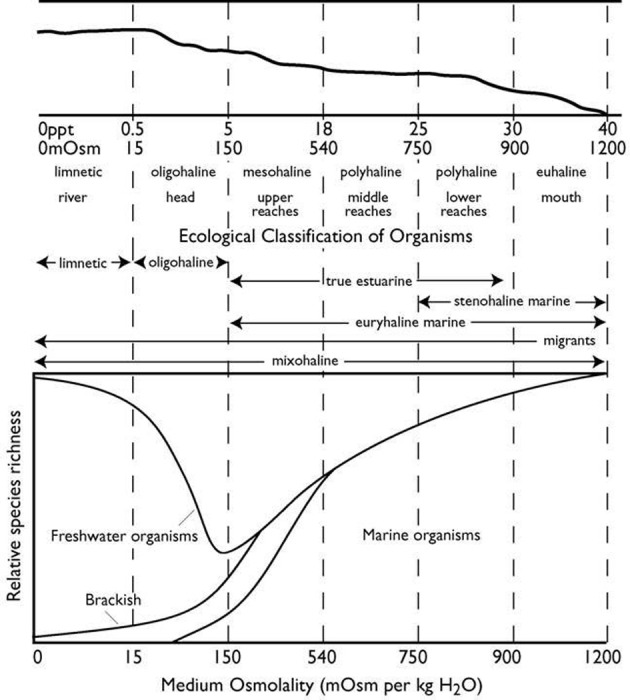
**Schematic representation of a typical coastal plain estuary. Top panel**: longitudinal profile of the estuary; the solid sloping line represents the division between the water column above and the sediment below. The longitudinal salinity gradient of the estuary runs from riverine fresh water of 0 ppt salinity at the far left to full-strength seawater of up to 40 ppt salinity at the far right. Salinity is also shown as mosm kg^−1^ water. Specific zones of the estuary are based on annual salinity variation. **Middle panel**: ecological classification of the distributions of organisms that spend either part or all their life cycle along the salinity gradient of the estuary. Physical and ecological classification based on the “Venice” system of Carriker (1967). **Bottom panel**: relative species richness (number of species) of different ecological classifications found across the salinity zones of the estuary [redrawn from Gainey and Greenberg (1977), for the distribution of benthic marine and estuarine species].

Euryhaline marine invertebrates can survive large fluctuations in environmental salinity, from 35 to near 0 ppt (1050 to near 0 mOsm kg H_2_O^−1^). By having evolved the ability to cope with low and variable salinity, these species have been able to invade and exploit the estuarine environment. Estuaries are broadly defined as bodies of water in which fresh water from rivers mixes with salt water from oceans or bays (Gross, 1972). There are a number of different classes of estuaries, but they all share one defining feature: a longitudinal salinity gradient that runs from just above 0 ppt (fresh water entry from a river) to as high as 40 ppt (full-strength sea water from the coastal ocean, bays, or lagoons) (Figure 1). Many estuaries, especially drowned river beds that occur primarily in coastal plains, can also be divided into discrete zones, based on annual variations in salinity (Figure 1). Furthermore, superimposed upon this gradient is a pattern of fluctuating salinity, resulting from tidal movements of sea water, seasonal changes in rainfall and fresh water runoff, and catastrophic dilutions from events such as tropical storms and hurricanes. As such, the estuary is a physically harsh environment that is low in number of competing species (Figure 1).

The abundance of marine species, which dominate in the estuarine zones of high salinity, drops off in the mesohaline zone (below 18 ppt), and overall species richness is lowest in the waters between 5 and 8 ppt, the border between the mesohaline and oligohaline zones (see also Khlebovich, 1969; Remane, 1971). Low and variable salinity thus represents a major physical-chemical barrier to the invasion of the most dilute waters of the estuary (Gainey and Greenberg, 1977; Deaton and Greenberg, 1986). The distribution of organisms within the estuary appears to coincide with the physical zones of the estuary as delineated by the salinity gradient. Stenohaline marine species, those with the narrowest salinity tolerance, are primarily restricted to the euhaline and lower polyhaline zones. True estuarine species and euryhaline marine species, those with a wider salinity tolerance, still do not survive below 5 ppt.

The ecological and evolutionary advantage of being able to survive in the estuary lies in the fact that estuaries are nutrient-rich environments. Estuarine circulation results in the entrainment of nutrients in the water column, similar to what occurs in cold water upwellings, making estuarine waters highly productive (Gross, 1972). Any species that can tolerate the physical challenges of the estuary can exploit a habitat that is rich in resources and low in competition. It is therefore reasonable to argue that there was significant selective pressure for the development of physiological and biochemical mechanisms for survival in low salinity. While this is true in general for all marine species, it has been extensively documented in the crustaceans.

## Mechanisms of salt and water balance

### Osmoconformers rely on isosmotic intracellular volume regulation

There are two fundamental physiological mechanisms present in marine organisms that are used to cope with changes in environmental salinity: (1) isosmotic intracellular volume regulation, and (2) anisosmotic extracellular osmoregulation, concepts first described in detail by Florkin and Schoffeniels (1969). These general mechanisms are also found in crustaceans. The overwhelming majority of marine crustacean species are osmotic and ionic conformers at full-strength seawater (see Mantel and Farmer, 1983, for a comprehensive review). Osmoconformers are characterized by extracellular fluid (i.e., hemolymph) osmotic and ionic concentrations that are slightly above those in the ambient medium, but which change in direct proportion to changes in ambient concentrations (e.g., Pierce, 1970). These organisms experience hemodilution and cellular water gain as a result of low salinity exposure, and undergo cell volume increases and resultant cell swelling. Furthermore, since almost all marine crustaceans, even those that are regulators below 26 ppt salinity, are osmoconformers in salinities between 35 and 26 ppt, all will experience a dilution of the hemolymph of at least 270 mOsm kg H_2_O^−1^. Below 26 ppt, osmoregulators turn on mechanisms of anisomotic extracellular regulation that stabilize hemolymph osmotic and ionic concentrations (see below), while osmoconformers experience further hemodilution. The compensatory mechanism to hemodilution and cell swelling involves a cell volume regulatory response, in which the intracellular osmotic concentration is reduced by reducing the concentration of the pool of intracellular osmolytes, such as inorganic ions (mostly K^+^) and small organic molecules (amino acids and quaternary ammonium compounds) (Pierce and Amende, 1981; Pierce, 1982; see also reviews by Strange, 1994; Henry, 1995). These compounds are released from the intracellular fluid intact, drawing osmotically obligated water with them, thus reducing cell volume. This mechanism is probably universal, being present in tissues of all marine invertebrates, both stenohaline and euryhaline. The cell volume response is variable among species, and the magnitude of this response confers a corresponding degree of euryhalinity on some species (e.g., Moran and Pierce, 1984). However, differences in the cell volume response do not define any trend in the evolution of adaptations to extremes of low and fluction salinity (i.e., there are large differences in euryhalinity both within and among taxonomic groups that exist along salinity gradients). Regardless, the majority of osmotic/ionic conformers are excluded from salinities below 8–10 ppt found in the upper reaches of the estuary (Kinne, 1971). Marine stenohaline crustaceans typically have a lower lethal salinity near 18 ppt [reviewed by Mantel and Farmer (1983)].

### Euryhaline crustaceans are osmotic and ionic regulators

The only euryhaline species that have the potential to traverse the entire range of salinity, from 35 ppt to fresh water (near 0 ppt), are the osmotic/ionic regulators. Osmoregulation involves maintaining the osmotic and ionic concentrations of the extracellular fluid (hemolymph) at concentrations different from the external water, most typically in dilute seawater. Hyper-regulation results in these concentrations being maintained above those in the ambient medium and is the most common form of regulation found in crustaceans. Marine crustaceans make the transition from osmoconformity to osmoregulation at a salinity of 26 ppt, at which point hemolymph osmolality begins to be actively maintained above that of the surrounding seawater (Henry, 2005). For salinities below 26 ppt, crustaceans can be classified as strong, moderate, or weak osmoregulators depending on the degree of low salinity tolerance and the magnitude of the hemolymph-seawater osmotic difference. The blue crab *Callinectes sapidus*, and the Chinese mitten crab, *Eriochier sinensis*, are perhaps the best examples of strong osmoregulating species. *C. sapidus* is found in nature in salinities from 40 to 0 ppt and can survive direct transfers from 35 to 0 ppt in the laboratory. The blue crab maintains a gradient of greater than 600 mOsm kg H_2_O^−1^ above ambient when acclimated to fresh water (Cameron, 1978a). The Chinese mitten crab migrates from seawater to freshwater as a juvenile and spends most of its adult life in riverine habitats. This species is also a very strong regulator, maintaining its hemolymph 550–700 mOsm above the ambient medium (Onken, 1999). A more moderate regulator is the green shore crab, *Carcinus maenas*, which is found in nature in salinities as low as 10 ppt and can survive laboratory acclimation to 8 ppt (Zanders, 1980). The green crab is able to maintain its hemolymph osmolality approximately 350 mOsm kg H_2_O^−1^ above ambient at 8 ppt. Interestingly, a weak osmoregulator is also member of the genus *Callinectes* (*C. similis*, the lesser blue crab). This species has a lower salinity limit near 15 ppt (Tagatz, 1967; Hsueh, 1992), suffers 80% mortality when gradually acclimated to 5 ppt in the laboratory, and maintains a maximum hemolymph-medium osmotic gradient of 250 mOsm kg H_2_O^−1^ (Engel, 1977). Species that spend the majority of their life cycles in fresh water can be either strong or moderate regulators. The blue crab and the Chinese crab, when in fresh water, maintain a hemolymph-water osmotic gradient of 600 mOsm or greater (see above). Other species, primarily crayfish, are more moderate regulators, displaying a hemolymph-medium osmotic gradient of 370–450 mOsm (e.g., Mantel and Farmer, 1983). Many of these species are actually quite stenohaline and are restricted to fresh water or water of very low salinity. More euryhaline fresh water species, such as the crayfish *Pacifastacus leniusculus*, make the transition between regulation and conformity at around 13 ppt (Wheatly and Henry, 1987).

## Form follows function: the cellular and ultrastructural features of branchial ion transporting epithelia

The structural basis of ion transport in crustacean gills lies in specialized adaptations at the cellular level. There are primarily two functional types of epithelia in the gill: a respiratory epithelium that is characterized by thin cells (1–2 μm thick), and an ion transporting epithelium characterized by thick cells (10–20 μm thick) (for two reviews, see Taylor and Taylor, 1992; Freire et al., 2008). These two types of cells typically have a heterogenous distribution among the gills, with the ion transporting thick cells being more concentrated in the posterior three pairs of gills. Early evidence from silver nitrate/silver chloride staining identified patches of ion transporting cells in the posterior gills, characterizing these gills as a mix of respiratory and ion-transporting cell types (e.g., Copeland and Fitzjarrell, 1968; Barra et al., 1983); the ion transporting patches are highly reduced or absent in anterior gills. Because this technique identifies cells that transport Cl^−^, the thick cells types were originally termed “chloride cells” in both crabs and fish (e.g., Zadunaisky, 1984). In euryhaline marine crabs acclimated to seawater, in which they are osmotic and ionic conformers, the ion-transporting patches are reduced in area, and the cells are concentrated in the lateral area of the gill near the afferent branchial vessel (Copeland and Fitzjarrell, 1968). In blue crabs acclimated to low salinity, the patch expands medially by as much as 4-fold to occupy a larger percent of the lamellar area (Neufeld et al., 1980); but even so, the patch still occupies only about 30% of the total lamellar surface area, according to measurements made in the green crab, *Carcinus maenas*, in low salinity (Péqueux et al., 1989; Compère et al., 1989). The process of proliferation takes on the order of 7–14 days, suggesting that it involves differentiation of unspecified, non-ion-transporting cells into chloride cells (Lovett et al., 2006).

The cells that make up the thick epithelium also have very distinct cellular and ultrastructural features that are congruent with the function of active ion transport. The basal membrane has varying degrees of infolding, presumably to increase the available surface area for transport; the same feature is found to a lesser extent on the apical membrane. Dense populations of mitochondria are found within these cells, leading to the more recent term “mitochondria-rich cells” (MRC). The mitochondria appear to be associated with the infoldings of the basal membrane in a manner that would facilitate their role in ion pumps. Rough endoplasmic reticulum, Golgi bodies, and microtubules are also defining features of this cell type (Copeland and Fitzjarrell, 1968; Neufeld et al., 1980; Taylor and Taylor, 1992; Freire et al., 2008). These cells also contain the highest concentrations and activity of the major transport proteins and enzymes that function in the ion transport mechanisms (detailed below). The term “ionocyte,” based on their function in ion transport, has also been used to describe these cells.

In freshwater species (e.g., crayfish) the ionocyte population is distributed among all gills, although the podobranch of the trichobranchiate, filamentous gills appears to have the highest concentration of ionocytes. Furthermore, individual filaments are either entirely respiratory or entirely ion-transporting (Dickson et al., 1991).

The mechanisms described below all take place in the ionocytes of the crustacean gill.

## Transbranchial NaCl transport in euryhaline crustacea: the tissue level

Transbranchial transport of NaCl in Crustacea is mainly related to osmotic regulation, although it has also been implicated in other branchial processes. For example, hemolymph acid-base regulation and nitrogen excretion are also believed to be coupled to the transport of Na^+^ and/or Cl^−^ (see other parts of this review). In the last hundred years, osmotic regulation in Crustacea has been studied on all organizational levels from whole animals to single proteins. Building on findings with whole animals, this part will focus on the tissue level. The biochemical and molecular levels will be addressed in the subsequent part. Previously, transbranchial NaCl transport and osmotic regulation in crustaceans has been reviewed on a regular basis (e.g., Potts and Parry, 1964; Smith and Linton, 1971; Kirschner, 1979, 1991; Mantel and Farmer, 1983; Péqueux et al., 1989; Lucu, 1990; Péqueux, 1995; Onken and Riestenpatt, 1998; Henry, 2001; Freire et al., 2008).

### Euryhaline crustaceans are hyper-regulators in low salinity

As outlined above, the vast majority of euryhaline crustaceans are hyperosmotic regulators that migrate between full-strength salinity of the open-ocean and estuaries containing brackish or fresh water. Consequently, the largest body of research consists of studies performed on active, transbranchial uptake of NaCl across the gills of these species. Accordingly, this section will emphasize the mechanisms of ion uptake that contribute to maintaining hemolymph osmolarity above that in the ambient medium. Only a few investigations have tried to analyze active NaCl secretion and its mechanisms in hypo-osmoregulating Crustacea (Green et al., 1959; Baldwin and Kirschner, 1976a; Evans et al., 1976; Martelo and Zanders, 1986; Luquet et al., 1997, 2002), and while this area deserves further study, there is not enough information currently to establish mechanisms of transport.

Based on studies with whole animals (cf. Potts and Parry, 1964; Mantel and Farmer, 1983; Kirschner, 1991), the principal adaptive strategies of hyperosmoregulating crustaceans are:

1. Reduction of the permeability of the body surface to salt and water.
2. Reduction of the osmotic gradient maintained across the body surface.
3. Increased production of urine to compensate for the passive inflow of water.
4. Active absorption of salt (mainly NaCl) to compensate for the passive, diffusive loss of salt (mainly NaCl).

The first two strategies reduce the passive flows of salt and water and, thus, make the second two strategies energetically less demanding. A quite comprehensive review of body surface permeabilities and osmotic and ionic concentrations in the hemolymph of many crustaceans acclimated to different media can be found in Mantel and Farmer (1983). The second two strategies actually compensate for the passive loss of salt and gain of water (via diffusion across the gills and bulk transport in urine) and maintain a more or less stable osmotic gradient across the body surface. Some freshwater species evolved mechanisms of active salt absorption in the antennal glands and, thus, have the ability to reduce salt loss by producing hypo-osmotic urine (Riegel, 1963, 1965, 1968). However, the vast majority of euryhaline crustaceans produce urine that is isosmotic with the hemolymph, resulting in a considerable salt loss (e.g., Cameron and Batterton, 1978a,b).

### Crustaceans can be grouped by the characteristics of their osmoregulatory mechanisms

Already early in the last century it became clear that the gills are the major site of active NaCl absorption in hyperosmoregulating Crustacea (Koch, 1934). Transbranchial mechanisms of NaCl absorption have been studied primarily in species that migrate between seawater and a variety of coastal environments like lagoons, intertidal zones, and estuaries and, ultimately, the freshwater of rivers and lakes. Two main groups can be distinguished among hyperosmoregulators: weak and strong hyperregulators. The differentiation between weak and strong hyperosmoregulators is based on the osmotic gradients that the animals can maintain across their body surface (see “Introduction”). Interestingly, hyperosmoregulating crustaceans seem to have evolved considerably different mechanisms of active NaCl absorption in their gills, and they display marked differences in the electrical conductance of their gill epithelia (see below). This led Onken and Riestenpatt (1998), Kirschner (2002), and Freire et al. (2008) to identify weak regulators with high epithelial conductance and one mechanism of NaCl absorption, and strong regulators with low epithelial conductance and another mechanism. However, this approach seems not to be accurate and oversimplifies the real situation of considerable diversity among hyperregulating Crustacea. For example, there are strong regulators like *Callinectes sapidus* and *Chasmagnathus granulata* that seem to absorb NaCl with transporters formerly attributed to weak regulators. Therefore, the present review represents a revision of this previous classification, and now distinguishes mechanisms of transport based on gills that display epithelia having either high conductance or low conductance, instead of classifying species as either weak or strong hyperosmoregulators.

## Methodological aspects

Most studies of transbranchial NaCl absorption have been performed with gills of large crustaceans like crabs, crayfish, and lobsters. The larger gills of these animals facilitate techniques to study transepithelial ion transport, and to some degree it can be assumed that smaller crustaceans have evolved identical or at least similar mechanisms. NaCl uptake across the gills of hyperosmoregulating crustaceans has been studied with a large variety of techniques, including different forms of microscopic observation as well as biochemical and molecular analyses. This part of the current review summarizes the results of measurements of transepithelial transport with isolated and perfused gills as well as with split gill lamellae and epipodites mounted in Ussing-type chambers.

### The advantage of studying transport in isolated gills

**Figure 2:**
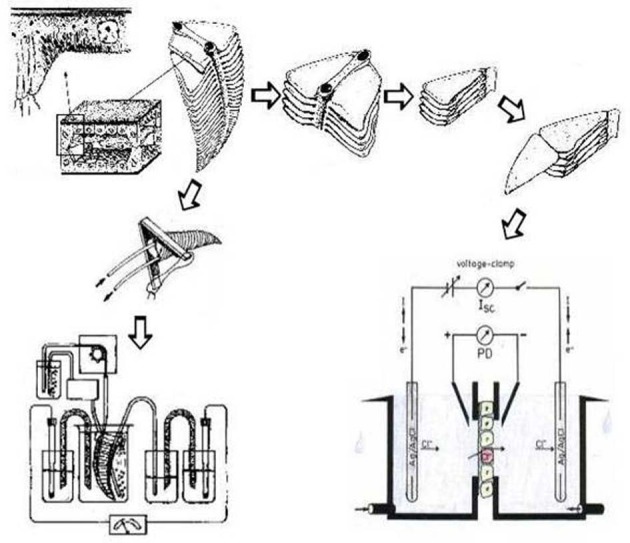
**The basic anatomical features of a crab gill (upper left), how isolated gills can be prepared and mounted for the measurement of transepithelial voltage and/or flues (lower left), and how crab gill lamellae are isolated and split for measurements in modified Ussing chambers (right).** Modified after Onken and Riestenpatt (1998) and Siebers et al. (1985).

Isolated gills of Crustacea survive for hours when maintained in aerated or oxygenated solutions with adequate osmolarity, ion composition, and nutrient content. Isolated gills can be used in different ways for transport studies (see Figure 2). Gills can be perfused in a way that the gill epithelium covered with cuticle on its apical side completely separates an external bathing medium and a hemolymph-side perfusate (e.g., Koch et al., 1954; Croghan et al., 1965; King and Schoffeniels, 1969; Péqueux and Gilles, 1978; Siebers et al., 1985). Such a preparation allows the measurement of transepithelial movements of ions, if radioactive tracers are applied. This can be done with the simultaneous addition of pharmacological inhibitors to study ion transport mechanisms directly (e.g., Burnett and Towle, 1990). In addition, the transepithelial voltage—a qualitative indicator for electrogenic movement of ions across the epithelium—can be monitored. The advantage of these techniques is that the transbranchial influx and efflux of ions can be quantified for a complete gill and standardized to the fresh weight of gill tissue. In order to facilitate the interpretation of the transepithelial voltage recorded with perfused gills, identical solutions must be used as bath and perfusate. Otherwise the voltage may consist of two components that are difficult to discriminate: a voltage that reflects active, electrogenic ion transport along the transcellular transport route, and a voltage related to paracellular diffusion of an ion species down its transepithelial concentration gradient. On the other hand, more advanced electrophysiological analyses that can be performed with planar epithelia (e.g., frog skin) are impossible with the complex structure of a whole perfused gill (cf. Figure 2). However, this disadvantage was overcome with gills that consist at least in part of planar structures like the lamellar gills of crabs, the sail-like epipodite of lobsters and crayfish, and the pleopods of isopods (Schwarz and Graszynski, 1989; Onken and Siebers, 1992; Lucu and Devescovi, 1999; Postel et al., 2000; Onken et al., 2003). Such planar preparations consist of the single-layered epithelium covered with cuticle on the apical side, which can be mounted in Ussing-type chambers. This allows for the quantitative measurement of electrogenic, transcellular transport as area-specific short-circuit current (*I*_*sc*_), as well as the simultaneous monitoring of the transepithelial conductance (*G*_*te*_). Taken together, these measurements allow for more advanced circuit analyses that can distinguish transcellular (*G*_*c*_) and paracellular (*G*_*p*_) conductances and determine transcellular electromotive forces, which reflect transmembrane electrochemical gradients or, ultimately, the power of the ion pumps that generate and maintain them. One of the studies with these small areas of gill tissue succeeded to simultaneously record short-circuit currents and radioactive tracer fluxes (Riestenpatt et al., 1996) in order to verify the equivalence of electrical current and net flux. Moreover, more sophisticated techniques like the measurement of membrane voltages with microelectrodes (Onken et al., 1991), or the determination of singler channel currents with current-noise analysis (Zeiske et al., 1992), were possible. A potential methodological problem that is not resolved by the use of planar preparations mounted in an Ussing-type chamber arises from the cuticle, which does not only reflect an additional barrier when compared to many other epithelial tissues, but also may interact or interfere with drugs that are used to identify membrane transporters. It has been observed, for example, that amiloride dramatically reduces conductance and Na^+^ flux across the cuticle of *Carcinus maenas* (Onken and Riestenpatt, 2002). Another disadvantage is the difficulty of simultaneously performing electrophysiological analyses and radioactive tracer fluxes. Because of the very small surface area that can be mounted in Ussing-type chambers, the radioactive doses must be quite high.

## Mechanisms of ion uptake

### High conductance versus low conductance epithelia

During the first 30 years after Ussing and collaborators (Ussing and Zerahn, 1951; Koefoed-Johnson and Ussing, 1958; Ussing, 1960) introduced the pathbreaking electrophysiological approach to study epithelial transport, electrophysiological parameters have been established that characterize transporting epithelia and allowed distinguishing different groups of transporting epithelia (see also Yonath and Civan, 1971). The two extreme cases are “leaky” and “tight” epithelia.

So-called “leaky” epithelia have a high electrical conductance. The ratio of their paracellular to transepithelial conductance (*G*_*p*_/*G*_*te*_-ratio) is high, often close to 1. Thus, the overall tissue conductance is mainly determined by the paracellular junctions. These epithelia can usually not maintain high electrochemical gradients, but they often generate very high transport rates like the proximal tubule of the mammalian kidney or the small intestine.

So-called “tight” epithelia, in contrast, have a low electrical conductance. Their *G*_*p*_/*G*_*te*_-ratio is low, often 0.1 or below. Because the transepithelial conductance (*G*_*te*_) is the sum of the transcellular (*G*_*c*_) and paracellular conductance (*G*_*p*_), the overall tissue conductance of tight epithelia is mainly determined by *G*_*c*_. Tight epithelia usually maintain high electrochemical gradients without necessarily generating high transport rates. Examples of tight epithelia are the amphibian skin, the distal part of the mammalian nephron, and the colon.

After Schwarz and Graszynski (1989) introduced the mounting of split gill lamellae in Ussing-type chambers, the characteristics of crustacean gill epithelia could be analyzed according to the standards established with other transporting epithelia during the decades before. These findings will be emphasized below. Three groups of crustaceans are distinguished in the following:

1. Euryhaline osmoconformers with very high conductance gills and no active transport.
2. Hyperosmoregulators with high conductance (“leaky”) gills and active NaCl uptake.
3. Hyperosmoregulators with low conductance (“tight”) gills and active NaCl uptake.

### Osmoconformers in seawater and moderately dilute seawater

All marine crustaceans appear to have the capability of intracellular osmoregulation, also called cell volume regulation (see also “Introduction”). This allows these animals to tolerate limited changes in their extracellular body fluid when they migrate between seawater and moderately diluted brackish waters (e.g., Gilles and Pequeux, 1981; Henry, 2001; Augusto et al., 2009).

The gill cuticle of osmoconforming crustaceans shows a higher conductance than the gill cuticle of hyperosmoregulators (Lignon, 1987; Lignon and Pequeux, 1990). When hemiepipodites of the marine, osmoconforming spiny lobster, *Palinurus elephas*, were analyzed in an Ussing-chamber, they showed very high transbranchial conductances (*G*_*te*_) of above 200 mS cm^−2^ (Lucu et al., 2000) and a very low short-circuit current (*I*_*sc*_). Neither *I*_*sc*_ nor *G*_*te*_ showed a significant change after transfer of the animals to a dilute medium. Another example is *Cancer pagurus*, for which a conductance of 250 mS cm^−2^ was determined (Weihrauch et al., 1999a,b). When split gill lamellae of osmoconforming Chinese crabs (*E. sinensis*) acclimated to seawater were studied in Ussing-chambers, the preparations did not show any active transport, and the transepithelial conductance was 7 mS cm^−2^. In the absence of active transport this conductance reflects ion movements via the paracellular junctions. Compared with the other osmoconforming animals, this conductance appears very low and may reflect the fact that Chinese crabs spend the largest proportion of their life as strong hyperregulators in freshwater. In hyperosmoregulating Chinese crabs acclimated to brackish water or freshwater, the paracellular conductance of the gill epithelium is 10–20 times lower (Onken, 1999, see also below).

Many euryhaline hyperosmoregulators are isosmotic in seawater above 26 ppt salinity (Henry, 2005), and in this respect they are physiologically similar to osmoconformers. In this situation net fluxes of Na^+^ or Cl^−^ are absent, and transepithelial voltages are absent or low. Essentially, the physiological mechanisms of active transport are silent at high salinity, and they are activated below the critical salinity of 26 ppt (see below). Osmoconformers, however, lack the ability to activate these mechanisms.

Altogether it can be summarized that the gills of marine, osmoconforming crustaceans do not show any active transport of NaCl, and the conductance is very high, indicating that Na^+^ and Cl^−^ diffuse passively and almost freely across the cuticle and epithelium of the gills. From an evolutionary point of view it seems evident that this osmoconforming behavior reflects the original form of the Crustacea. However, it is noteworthy to mention that a number of species are considerably hypo-osmotic when in seawater, and it cannot be excluded that the gills of these animals are engaged in active NaCl secretion. This active NaCl secretion in crustacean hypo-osmoregulators is so far not sufficiently studied and must be addressed in future investigations.

### Hyperosmoregulators in dilute seawater and freshwater

All hyperosmoregulating crustaceans actively absorb NaCl across their gills and maintain their hemolymph osmolarity above the osmolarity of the medium (see above). In freshwater crayfish, all gills are engaged in active NaCl absorption, and these animals can reduce salt loss by producing dilute urine (see also above). In contrast, the posterior gills are the primary site of active NaCl uptake in euryhaline marine crabs, and these animals can lose considerable amounts of salt via urine that is isosmotic with the hemolymph. The anterior gills of crabs are primarily engaged in respiration and excretion (cf. Mantel and Farmer, 1983; Péqueux, 1995; McNamara and Lima, 1997; Onken and Riestenpatt, 1998; Freire et al., 2008, see also below). In this part of the current review we exclusively address NaCl transporting gills.

### Common ground in low and high conductance gill epithelia

All hyperosmoregulators maintain an osmotic gradient by active NaCl uptake (see above). The basolateral membranes of all NaCl absorbing epithelia are usually equipped with three transporters: Na^+^/K^+^-ATPase, K^+^ channels, and Cl^−^ channels (see Figures 4 and 6). In the gills of hyperosmoregulating Crustacea, low conductance or high conductance, all three transporters have been demonstrated with specific inhibitors like ouabain (Na^+^/K^+^-ATPase), barium or cesium ions (K^+^ channels), and DPC or NPPB (Diphenylamine-2-carboxylic acid, 5-nitro-2-[3-phenylpropylamino]-benzoic acid; Cl^−^ channels). Addition of these drugs to the internal perfusion saline of isolated gills or to the internal bath of planar gill preparations mounted in an Ussing-type chamber resulted in decreased or abolished fluxes, transepithelial voltages, or short-circuit currents (Drews and Graszynski, 1987; Siebers et al., 1987; Onken and Graszynski, 1989; Onken et al., 1991, 2003; Pierrot et al., 1995a,b; Riestenpatt et al., 1996; Lucu and Devescovi, 1999; Postel et al., 2000; Luquet et al., 2002; Lucu et al., 2009; Lucu and Towle, 2010). For the Na^+^/K^+^-ATPase, the localization in the basolateral membrane has also been confirmed in histochemical studies (Towle and Kays, 1986; McNamara and Torres, 1999; Towle et al., 2001).

### High conductance gill epithelia: high rates of uptake compensate for salt loss

After adaptation to dilute ambient salinity, the transepithelial conductances of the gill epithelia of *Carcinus maenas*, *Chasmagnathus granulata*, *Homarus gammarus*, *Homarus americanus*, and *Idotea baltica* amounted to 40–80 mS cm^−2^ (Riestenpatt et al., 1996; Lucu and Devescovi, 1999; Postel et al., 2000; Onken et al., 2003; Lucu and Towle, 2010). After blocking the electrogenic, transcellular pathway of *Carcinus maenas* gills with Ba^2+^ or Cs^+^, Riestenpatt et al. (1996) found a reduction of the transepithelial conductance by about 50%. Thus, the *G*_*p*_/G_*te*_-ratio of about 0.5 characterizes the gills of *Carcinus* as moderately leaky. In *Chasmagnathus granulata*, the inhibition of the transcellular pathway was not followed by a significant conductance decrease at all (Onken et al., 2003). Consequently, the *G*_*p*_/G_*te*_-ratio is close to 1 and the gill epithelium of these animals is quite leaky. All animals of this group can be expected to lose considerable quantities of NaCl across the large surface of their gills and by producing isosmotic urine (see above). Based on high efflux values from experiments with whole animals or isolated gills, other crustaceans with high conductance gills are most likely *Callinectes sapidus* (Cameron, 1978a,b), *Cancer magister* (cf. Kirschner, 1991), *Pachygrapsus marmoratus* (Pierrot et al., 1995a,b), *Uca pugilator and Uca pugnax* (Baldwin and Kirschner, 1976b). Another indicator for membership in this group of hyperosmoregulating crustaceans can be the relatively low transepithelial voltage (below 15 mV) typical for leaky epithelia. Based on the large loss of salt in dilute media, these crustaceans must evidently invest considerable amounts of energy in active transport/uptake of salt in order to compensate for the large rates of diffusive salt loss. It is therefore not surprising that gill preparations from these animals showed high transport rates (150–450 μA cm^−2^, Riestenpatt et al., 1996; Lucu and Devescovi, 1999; Postel et al., 2000; Onken et al., 2003; Lucu and Towle, 2010) and a substantial increase of oxygen consumption after adaptation to dilute ambient media (Siebers et al., 1982; Lucu and Pavičić, 1995; Piller et al., 1995).

**Figure 3:**
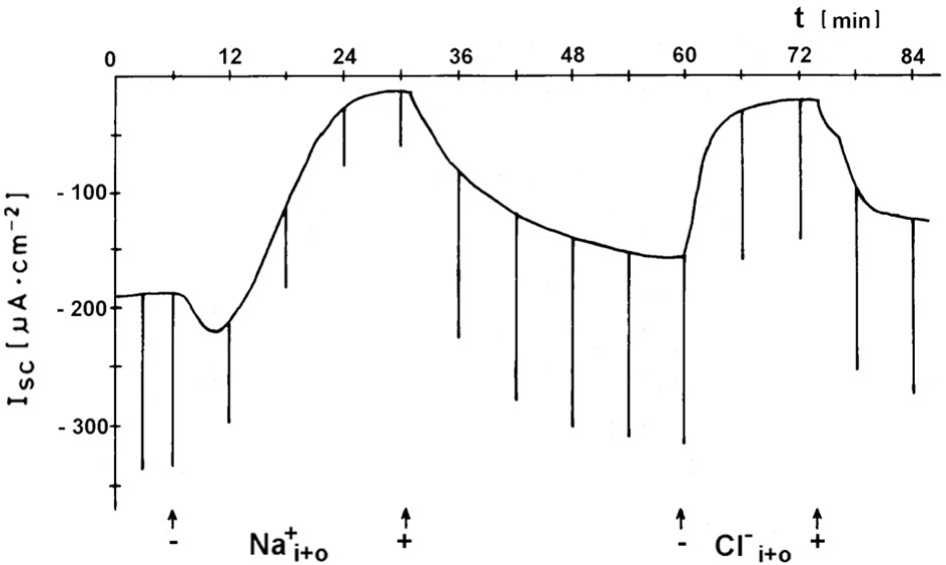
**Coupled absorption of NaCl in hyperosmoregulating species characterized by “leaky” gill epithelia.** Representative time-course of the short-circuit current (I_*sc*_), showing the effects of either Na^+^ substitution (choline) or Cl^−^ substitution (gluconates) on both sides of a split gill lamella of the shore crab *Carcinus maenas*. Current deflections are due to 5 mV voltage pulses and are directly proportional to the transepithelial conductance (from Onken and Siebers, 1992).

Active transbranchial NaCl uptake in *Carcinus maenas* (Siebers et al., 1987), *Chasmagnathus granulata* (Luquet et al., 2002; Onken et al., 2003), *Pachygrapsus marmoratus* (Pierrot et al., 1995a,b), *Uca tangeri* (Drews and Graszynski, 1987), *Homarus gammarus* (Lucu and Devescovi, 1999; Lucu et al., 2009), *Homarus americanus* (Lucu and Towle, 2010), and *Idotea baltica* (Postel et al., 2000) completely depended on a functioning Na^+^/K^+^-ATPase. Perfusion of gill epithelium suggested that apically located cotransport mechanisms of Na^+^ and Cl^−^ influxes were linked with energetics of Na/K-ATPase activity (Lucu and Siebers, 1987). When added to the internal medium of perfused gills or planar preparations, ouabains abolished influxes, transepithelial voltage, and short-circuit currents. No other plasma membrane ion pump seems to be involved in active NaCl absorption. Using ion substitutions, it was shown that NaCl uptake proceeds in a coupled mode (Onken and Siebers, 1992; Onken et al., 2003; Lucu and Towle, 2010, see Figure 3). Simultaneously studying fluxes of radioactive tracers and short-circuit current with split gill lamellae of the shore crab *Carcinus maenas*, Riestenpatt et al. (1996) demonstrated that NaCl uptake also depends on apical Na^+^/K^+^/2Cl^−^ cotransporters. Under short-circuit conditions, the ratio of the net influxes of Na^+^ and Cl^−^ was found to be 1:2 as is predicted by the stoichiometry of Na^+^/K^+^/2Cl^−^ cotransporters. Because specific inhibitors of this transporter (bumetanide or furosemide) apparently do not cross the cuticle, Riestenpatt et al. (1996) used a very elegant approach to demonstrate the Na^+^- and K^+^-dependence of the apical Cl^−^ transporters. After functionally eliminating the basolateral membrane of split gill lamellae with an ionophore (amphotericin B), an inward-directed Cl^−^ gradient was established, and Cl^−^ influxes were measured in the presence and absence of external Na^+^ and/or K^+^. The results confirmed the Na^+^ and K^+^ dependent character of the apical Cl^−^ transporter. For *Carcinus maenas* and *Callinectes sapidus*, these transporters were later also found using molecular techniques (Towle and Weihrauch, 2001, see also below). NaCl uptake via apical Na^+^/K^+^/2Cl^−^ cotransporters requires apical K^+^ channels to supply the cotransporters with potassium from the cell interior, to hyperpolarize the cell (helping to drive Cl^−^ ions from the cell to the hemolymph-side through basolateral Cl^−^ channels), and to give the overall transport an electrogenic character (generating an outside positive transepithelial voltage used to drive Na^+^ ions across the paracellular junctions, and to guarantee that transepithelial NaCl absorption is equimolar). The presence of these apical K^+^ channels has been demonstrated in electrophysiological studies with Cs^+^ ions as blockers in the external bath of planar preparations mounted in Ussing-chambers (Riestenpatt et al., 1996; Onken et al., 2003; Lucu and Towle, 2010).

**Figure 4:**
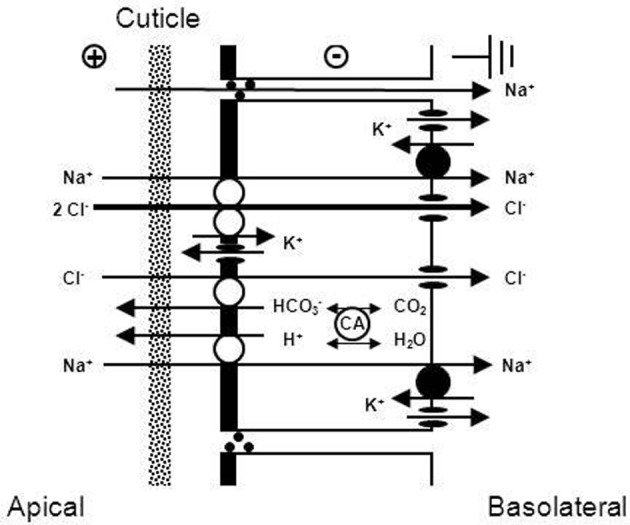
**Transport mechanism proposed for transbranchial NaCl uptake across the gills of hyperosmoregulators characterized by “leaky” gill epithelia and high transport rates (from Onken et al., 2003).** Active NaCl uptake is coupled and proceeds via Na^+^/K^+^/2Cl^−^ cotransporters in the apical membrane. Absorbed Na^+^ ions are then pumped by the Na^+^/K^+^-ATPase across the basolateral membrane. The presence of basolateral and apical K^+^ channels results in a negative electrical potential inside of the cells which drives the movement of Cl^−^ ions through Cl^−^ channels in the basolateral membrane. Apical K^+^ channels are also of importance to supply the apical Na^+^/K^+^/2Cl^−^ cotransporters with K^+^ ions, and give the overall transport its electrogenic nature. The outside positive transbranchial voltage can then be used as the driving force for paracellular Na^+^ absorption to ensure equimolar NaCl absorption. In addition, active NaCl uptake proceeds via cation (Na^+^/H^+^) and anion (Cl^−^/HCO^−^_3_) exchangers in the apical membrane, supported by a carbonic anhydrase that catalyzes the fast production of acid/base equivalents as intracellular substrates for the exchangers.

A number of other studies (Lucu, 1990; Onken et al., 2003; Henry, 2001; Henry et al., 2002, 2006) indicated that variable amounts of NaCl are absorbed via apical Na^+^/H^+^ and Cl^−^/HCO^−^_3_ exchangers linked by carbonic anhydrase (CA), which rapidly supplies the exchangers with their acid/base substrates (see Figure 4). Effects of external amiloride or DIDS (inhibitors of Na^+^/H^+^ and Cl^−^/HCO^−^_3_ exchangers, respectively) and of acetazolamide (inhibitor of CA) on tracer fluxes or transepithelial voltages across isolated gills have been reported a number of times (cf. Lucu, 1990). In *Chasmagnathus granulata*, acetazolamide inhibited about 10–20% of Na^+^ influx without affecting the transepithelial voltage, indicating that a part of transcellular NaCl transport is electroneutral and possibly proceeds via apical Na^+^/H^+^ and Cl^−^/HCO^−^_3_ exchangers (Onken et al., 2003). In whole-animal studies using *Callinectes sapidus*, acclimated to freshwater, Cameron (1979) observed that injected acetazolamide (100 μmol/L) abolished the net influxes of Na^+^ and Cl^−^ with no effect on the electrical voltage across the body surface of the animals, suggesting that a major proportion of active NaCl absorption may proceed via Na^+^/H^+^ and Cl^−^/HCO^−^_3_ exchangers. Treatment with acetazolamide also resulted in Na^+^ and Cl^−^ concentrations being lowered in *Pachygrapsus crassipes*, *Callinectes sapidus*, and *Carcinus maenas*, acclimated to low salinity in which they are osmoregulating (Burnett et al., 1981; Henry and Cameron, 1983; Henry et al., 2003). Furthermore, treatment of *C. sapidus* with acetazolamide (100 μmol/L) during the acute phase of low salinity acclimation results in 100% mortality as a result of the breakdown in the ability to maintain hemolymph ion concentrations above those in the medium (Henry and Cameron, 1982b). Moreover, increased activity and expression of CA in the gills of *Callinectes sapidus* and *Carcinus maenas*, after acclimation to dilute ambient media corroborates the presence of a CA-dependent component of active NaCl uptake (Henry and Cameron, 1982a; Henry, 2001; Henry et al., 2002, 2003, 2006). It could well be that the two mechanisms of active NaCl uptake, one electrogenic and the other electroneutral, are variable features of different species or populations.

In summary, hyperosmoregulators with high conductance gill epithelia suffer from high losses of NaCl when in dilute ambient media. The gill epithelia of these animals generate high rates of active NaCl uptake at rather low transepithelial voltages. The mechanisms of active NaCl uptake resemble those of other well-studied, high conductance epithelia like the proximal tubule and the thick ascending limb of Henle's loop of the mammalian kidney (Greger and Kunzelmann, 1990).

### Low conductance gill epithelia: emphasis on prevention of salt loss

With regard to the basic epithelial characteristics of representatives of this group, the most reliable data are available for Chinese crabs, *Eriocheir sinensis*, acclimated to freshwater. Transepithelial voltages of at times far above 50 mV were recorded with isolated Chinese crab gills (Gilles and Péqueux, 1985, 1986; Péqueux and Gilles, 1988; Onken and Graszynski, 1989), indicating the ability of the gills to generate and maintain high electrochemical gradients. The conductances of the paracellular junctions (*G*_*p*_) and the transcellular pathway (*G*_*c*_) were found to be 0.75 mS cm^−2^ and 4 mS cm^−2^, respectively (Onken, 1999), characterizing the epithelium as tight. Another representative of this group is the red freshwater crab, *Dilocarcinus pagei*. The gill lamellae of these animals show an interesting asymmetry with a thin, distal epithelium and a thick, proximal epithelium within each lamella (Onken and McNamara, 2002). The distal epithelium had an average transepithelial conductance of about 4 mS cm^−2^ which dropped to about 1.5 mS cm^−2^ when Cl^−^ was substituted for nitrate to stop transcellular transport in this preparation. Consistent with tight epithelia, both *Eriocheir* and *Dilocarcinus* have relatively low transport rates (below 100 μA cm^−2^ without hormonal stimulation). Conductance measurements with the gills of other freshwater crabs or crayfish are not yet available. However, such animals are most likely members of this group as they maintain very high concentration gradients across their body surface. In any case, the major strategy of hyperosmotic regulation in animals with low conductance gills is to avoid loss of salt by forming a tight barrier to the very dilute environment. This reduced salt loss is evidently of benefit, because it reduces the amount of energy expended for active transport against the large gradient. Morphological studies on the potential changes that occur at the apical membrane of the gill will greatly contribute to the model of low permeability in the gill epithelia of frershwater crustaceans.

### The mechanism of ion transport in low-conductance gills

**Figure 5:**
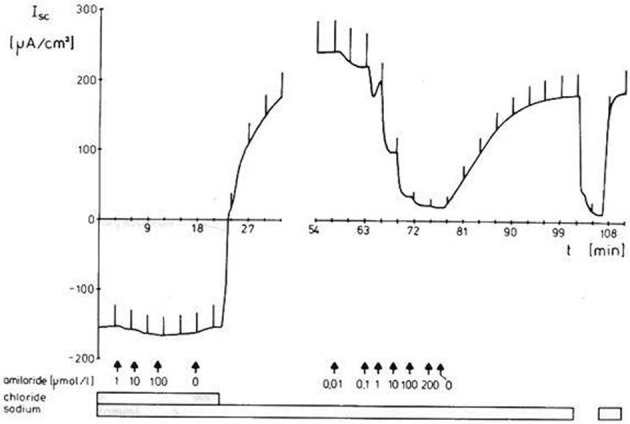
**Independent absorption of Na^+^ and Cl^−^ in a hyperosmoregulators characterized by a low conductance (“tight”) gill epithelium.** Representative time-course of the short-circuit current (I_*sc*_), showing the negative I_*sc*_ [reflecting active and Na^+^-independent Cl^−^-absorption; further characterized in Onken et al. (1991)] and the positive I_*sc*_ [reflecting Cl^−^- independent Na^+^-absorption; further characterized in Zeiske et al. (1992)] across a split gill lamella of the Chinese crab *Eriocheir sinensis*. Current deflections are due to 10 mV voltage pulses and are directly proportional to the transepithelial conductance (from Zeiske et al., 1992).

The exploration of the mechanisms of active NaCl absorption in hyperosmoregulators began with the invasion of northern European waters by the Chinese crab, *Eriocheir sinensis*. These animals reproduce in the sea but spend almost their entire life in freshwater, often migrating vast distances upstream. In freshwater, the animals maintain an osmotic gradient of about 600 mosmol kg^−1^, which is about double the gradient of freshwater fish or amphibia. August Krogh (1938) used these animals as part of his groundbreaking study about “The active absorption of ions in some freshwater animals.” Working with intact, living Chinese crabs, Krogh determined that the absorption of Na^+^ and Cl^−^ is independent of each other and proceeds via exchange of Na^+^ ions for protons (H^+^) and of Cl^−^ ions for bicarbonate (HCO^−^_3_). In the following 70 years, active NaCl absorption in Chinese crabs was investigated in numerous studies on the level of whole animals, isolated gills, split gill lamellae, single proteins, using a broad spectrum of experimental techniques, only to come to basically the same conclusion. Consistent with Krogh's findings, it was shown that the gill epithelium of Chinese crabs generates positive and negative short-circuit currents that reflect active absorption of Na^+^ and Cl^−^, respectively (Onken et al., 1991; Zeiske et al., 1992, see Figure 5).

**Figure 6:**
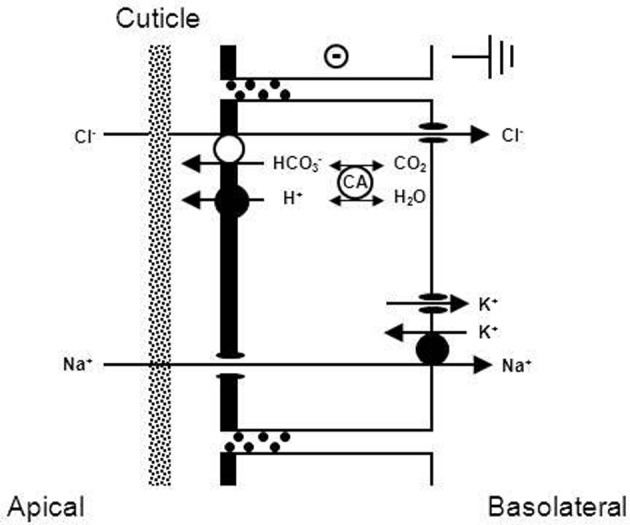
**Transport mechanism proposed for transbranchial NaCl absorption across the gills of hyperosmoregulators characterized by a low conductance (“tight”) epithelium (after Onken and Riestenpatt, 1998).** Active, Cl^−^ independent Na^+^ absorption proceeds via epithelial, amiloride-sensitive Na^+^ channels (ENAC) in the apical membrane and the Na^+^/K^+^-ATPase in the basolateral membrane. Active, Na^+^ independent Cl^−^ absorption proceeds via apical Cl^−^/HCO^−^_3_ exchangers and basolateral Cl^−^ channels. Cytoplasmic carbonic anhydrase (CA) supports both Na^+^ and Cl^−^ transport. Basolateral K^+^ channels generate a negative electrical potential in the cells. An apical V-type H^+^-pump increases intracellular HCO^−^_3_, supporting transapical Cl^−^ absorption. Moreover, the electrogenic nature of the H^+^-pump hyperpolarizes the intracellular negativity, supporting the entry of Na^+^ ions across the apical membrane and the exit of Cl^−^ ions across the basolateral membrane.

With regard to the mechanisms of NaCl absorption across Chinese crab gills, split gill lamellae mounted in modified Ussing-chambers offered the deepest insight. Initiated by a study that showed that crab gill lamellae can be split without major damage (Schwarz and Graszynski, 1989), a series of investigations followed that uncovered the mechanism shown in Figure 6. Onken et al. (1991) showed that such split gill lamellae generate a negative short-circuit current in the absence of external Na^+^. This current depended on external Cl^−^, was inhibited with external DIDS/SITS and internal DPC, but was unaffected by internal ouabain. Thus, Na^+^-independent Cl^−^ absorption seems to proceed via apical Cl^−^/HCO^−^_3_ exchange and basolateral Cl^−^ channels and is independent of the Na^+^/K^+^-ATPase. These findings demanded an electrogenic pathway in the apical membrane to explain the electrogenic nature of the transport, an ATP-consuming mechanism to explain the active nature of the transport, and a transporter that eliminates protons from the cell in order to keep the cellular pH stable at the continuous secretion of bicarbonate. An apical H^+^-pump was proposed, and it was identified in the apical membrane a few years later (Onken and Putzenlechner, 1995). The acid/base equivalents needed as substrates for the apical transporters are rapidly supplied by a cytoplasmic CA (Onken et al., 1991) that shows increased activities when the animals are acclimated to freshwater (Olsowski et al., 1995). In another study (Zeiske et al., 1992), split gill lamellae of Chinese crabs were shown to generate a positive short-circuit current in the absence of external Cl^−^. This current was Na^+^-dependent and was inhibited with external amiloride and internal ouabain. Amiloride-induced current-noise analysis revealed that the amiloride-sensitive transporter is, indeed, an epithelial Na^+^ channel (Zeiske et al., 1992), and not an effect on the gill cuticle (cf. Onken and Riestenpatt, 2002). Under more *in vivo*-like conditions, transcellular absorption of Na^+^ and Cl^−^ are electrically coupled, guaranteeing equimolar absorption of NaCl. Moreover, at increased external NaCl concentrations the apical Na^+^ channels close and NaCl absorption is rapidly decreased, because electrical coupling between Na^+^ and Cl^−^ then involves movement of Na^+^ along the low conductance paracellular pathway (Onken, 1999).

Taking apical, amiloride-sensitive Na^+^ channels or apical V-type H^+^-pumps as indicators for the mechanism of NaCl absorption in low conductance gill epithelia, evidence for the presence of this, or a very similar mechanism, has been found in freshwater crayfish (Kirschner et al., 1973; Ehrenfeld, 1974; Zare and Greenaway, 1998; Zetino et al., 2001; Kirschner, 2002) and in the hololimnetic crab *Dilocarcinus pagei* (Onken and McNamara, 2002; Weihrauch et al., 2004). *Chasmagnathus granulata* has high conductance gills (see above). However, it appears that the above described mechanism of NaCl absorption (see Figure 6) plays some role when the animals are acclimated to very low salinities (Genovese et al., 2005). Using antibodies, Tsai and Lin (2007) observed V-ATPases in apical membranes of the gills of *Eriocheir sinensis* and four other crab species from the Ocypodidae and Grapsidae families, whereas the V-ATPases were located in the cytoplasm in some other species (for a possibly function of cytoplasmic V-ATPase see also below in the nitrogen excretion section).

In summary, crustaceans with low conductance gill epithelia have reduced salt loss in very dilute ambient media. These animals can maintain very high electrochemical gradients across their body surface at rather low rates of active absorption. It is noteworthy to mention here that the energetic cost of ion transport depends on the magnitude of the gradient against which the active transport precedes. Evidently, most animals that spend long time periods in freshwater reduce the loss of salt by structural adaptations in order to avoid the high energy requirement of active transport against the very large concentration gradient. The mechanisms of active NaCl absorption resemble those of other well-studied, low conductance epithelia like the frog skin (Ehrenfeld and Klein, 1997), or the distal tubule and the collecting duct of the mammalian kidney (e.g., Larsen, 1988; Evans et al., 2005). A similar mechanism has also been proposed for freshwater fish as well (Goss et al., 1992).

## Regulation of transport rates

When euryhaline marine crustaceans make the transition from osmoconformity to osmoregulation, typically at or near a salinity of 26 ppt (Henry, 2005), the mechanisms of active transbranchial ion uptake, which are silent at high salinity, are activated (Cameron, 1978a,b; Lucu and Siebers, 1986; Péqueux et al., 1989; Péqueux, 1995; Onken, 1999). In addition to a simple, salinity-mediated turning on of these processes, active ion transport has been shown to be modulated by hormonal and non-hormonal factors as well. In gills of *Eriocheir sinensis* and *Chasmagnathus granulata*, changes in the hemolymph-side osmolarity acted as strong modulators of transport rates (Onken, 1996; Tresguerres et al., 2003). Reduced osmolarity resulted in stimulation of NaCl uptake, whereas increased osmolarity reduced it. Because these results were obtained with isolated gills, this appears to be a mechanism of autoregulation and is evidently hormone-independent.

There is also evidence that hormonal factors can regulate NaCl uptake in crabs already in the osmorgulating state. An unidentified hemolymph factor in *Callinectes sapidus* was reported to increase branchial Na^+^/K^+^-ATPase activity, and thus presumably increase Na^+^ transport in gills in crabs in low salinity (Savage and Robinson, 1983). Also, stimulation of cyclic-AMP-dependent protein kinase A stimulated the Na^+^/K^+^-ATPase and increased Na^+^/Ca^2+^ exchange in the gills of *Carcinus maenas* (Lucu and Flik, 1999). A factor from the eyestalks stimulated active ion uptake across split gill lamellae of Chinese crabs via a cAMP- and PKA-mediated mechanism that resulted in increasing the number of apical Na^+^ channels and activating the apical V-ATPase (Riestenpatt et al., 1994; Onken et al., 2000). In *Pachygrapsus marmoratus*, crustacean hyperglycaemic hormone (CHH) was shown to stimulate Na^+^/K^+^-ATPase activity and thus increase transbranchial NaCl transport (Eckhardt et al., 1995; Spanings-Pierrot et al., 2000). Also dopamine, which was associated with the pericardial organs and seems to act via cAMP, was shown to increase active uptake in hyperosmoregulating crustaceans by stimulation of Na^+^/K^+^-ATPase (Sommer and Mantel, 1988; Morohashi et al., 1991; Mo et al., 1998; Halperin et al., 2004). Hormonal regulation in crustaceans has been a sparsely studied field that deserves more systematic investigation. The molecular basis of regulation is treated in detail below.

## Biochemical and molecular basis of branchial ion transport

### Enzymatic contribution to branchial ion transport

Branchial ion uptake in osmoregulating crustaceans is accomplished by the coordinated action of a suite of transport proteins and transport-related enzymes. Two of the transport-related enzymes that have been the most extensively studied on the biochemical level are the Na/K-ATPase and CA. The Na/K-ATPase was discovered in neurons of the crustacean, *Carcinus maenas* (Skou, 1957), but it was viewed as a cellular, trans-membrane transport enzyme that functioned in the generation of the resting potential in excitable cells. The Na/K-ATPase was first shown to be a trans-epithelial transport enzyme in fish gills (for an extensive review, see Evans et al., 2005). For crustaceans, the Na/K-ATPase was first shown to be a critical component of low salinity adaptation by Towle et al. (1976), in a study on the blue crab, *Callinectes sapidus*. The initial quantitative evidence for the role of CA in crustacean branchial ion transport was reported shortly thereafter, again in *C. sapidus* (Henry and Cameron, 1982a).

### Biochemical characteristics of transport-related enzymes

Biochemical studies based on these two enzymes have shown that transport-related enzymes share a number of characteristics that help define their physiological function. While some transport proteins are species- and tissue-specific, the Na/K-ATPase and CA have a universal distribution among gills of crustaceans. However, both enzymes are present in the gills of euryhaline crustacean species in higher activity than in non-branchial tissue (e.g., Towle et al., 1976; Henry and Cameron, 1982a,b; Siebers et al., 1982; D'Orazio and Holliday, 1985; Wheatly and Henry, 1987; Bottcher et al., 1991; Bouaricha et al., 1991; Henry, 1991; Lucu and Flik, 1999), and activities of both enzymes are higher in the gills of euryhaline species than in those of stenohaline, osmoconforming species (McDonough Spencer et al., 1979; Henry and Cameron, 1982a; Henry, 1984; Harris and Bayliss, 1988). Furthermore, in euryhaline marine crustaceans, both enzymes have a heterogenous distribution among the gills. Gills in euryhaline decapod crustaceans are typically specialized for either passive respiratory gas exchange or active ion transport. The respiratory gills are the anterior 4–6 gill pairs, depending on species, and these gills are characterized by a thin (0.1–1.0 μm) epithelium (Aldridge and Cameron, 1979), and low activities of both transport enzymes (Neufeld et al., 1980; Henry and Cameron, 1982a; Holliday, 1985; Bottcher et al., 1990a). Ion transporting gills are typically the posterior 2–3 pairs, have a thick (10–20 μm) epithelium, contain high activities of CA and the Na/K-ATPase, and possess dense populations of ion-transporting mitochondrial rich “chloride” cells (described in detail above). In freshwater crabs (e.g., *Dilocarcinus pagei*), both the respiratory and ion-transporting epithelia are thicker (2–5 μm and 18–20 μm, respectively), which could reflect lower overall gill permeability in freshwater-adapted species (Weihrauch et al., 2004). The posterior gills still have about 3.5 times higher activity of the Na/K-ATPase than do the anterior gills. In other freshwater crustacean species (e.g., crayfish), the respiratory and ion-transporting cells, and consequently the activities of CA and the Na/K-ATPase, are homogeneously distributed among all gills (Wheatly and Henry, 1987; Dickson et al., 1991), although they are heterogeneously distributed within the filaments of the trichobranchiate gills (see above).

### Enzyme activity is altered by environmental salinity

The signature feature of these transport-related enzymes, however, is that their activity is salinity-sensitive. Activities of the Na/K-ATPase and CA increase in the posterior, ion-transporting gills, during low salinity acclimation, of virtually all euryhaline, osmoregulating marine crustaceans studied (e.g., Towle et al., 1976; Henry and Cameron, 1982a,b; D'Orazio and Holliday, 1985; Holliday, 1985; Wheatly and Henry, 1987; Bouaricha et al., 1991; Henry et al., 2002, 2003; Lovett et al., 2007; Roy et al., 2007; Tsai and Lin, 2007). In the two species studied, the initial induction of activity occurs at the critical salinity at which the crabs make the transition from osmoconformity to osmoregulation, 26 ppt (Henry, 2005). Enzyme activity has been shown to increase approximately 4-fold for the Na/K-ATPase, and up to 20-fold for CA, depending on species, magnitude of low salinity decrease, and time of acclimation (Towle et al., 1976; Henry and Cameron, 1982a,b; Towle, 1984, 1997; Holliday, 1985; Lucu and Flik, 1999; Henry and Watts, 2001; Henry et al., 2002, 2003; Henry, 2005; Roy et al., 2007; Torres et al., 2007; Lucu et al., 2008). These increases are correlated to parallel increases in the population of mitochondria-rich “chloride” cells that occur in the ion-transporting gills in response to low salinity exposure (Neufeld et al., 1980; Lovett et al., 2006).

### Enzyme induction is needed to keep up with increased rates of ion transport

Freshwater and terrestrial species tend to have more stable activity levels for the Na/K-ATPase when exposed to salinity changes (Tsai and Lin, 2007). The differences in enzyme induction may lie in the relative permeabilities of the gills of marine vs. freshwater crustaceans. Marine species, such as the blue crab, *Callinectes sapidus*, have high permeability to salt and water in high salinity in which it is an osmoconformer, and this property is retained when the crab migrates into low salinity. High branchial permeability results in correspondingly high rates of diffusive salt loss through the gills: 344 μmol kg^−1^ hr^−1^ for Na^+^ efflux and 569 μmol kg^−1^ hr^−1^ for Cl^−^ efflux in the blue crab acclimated to fresh water (Cameron, 1978a). Branchial permeability and salt loss is comparatively low in freshwater species: the freshwater crayfish, *Astacus leptodactylus*, has a rate of branchial diffusive Na^+^ loss that is approximately half that of *Callinectes* (136 μmol kg^−1^ hr^−1^) (Ehrenfeld, 1974). Compounding branchial salt loss is the fact that marine crustaceans produce an isosmotic/isotonic urine, even when in dilute salinity. Again, in the blue crab, urinary salt loss is 239 and 255 μmol kg^−1^ hr^−1^ for Na^+^ and Cl^−^, respectively, accounting for 41% of the total salt loss from the crab's extracellular fluid (Cameron and Batterton, 1978a,b). In contrast, freshwater species produce a hypo-osmotic/hypo-tonic urine through the reabsorption of ions via active transport mechanisms located in the antennal gland. In the crayfish, *A. fluviatilis*, urinary salt loss accounts for only 5–8% of the total salt lost from the animal (Bryan, 1960). Freshwater crayfish (e.g., *Pacifasticus leniusculus*) also have high levels of activity of both the Na/K-ATPase and CA in gills and antennal gland (Wheatly and Henry, 1987; Henry and Wheatly, 1988).

The high rate of ion loss (both branchial and urinary) that marine crustaceans experience when exposed to low salinity is compensated for by increases in the rates of active ion uptake across the gills (see above). The induction of Na/K-ATPase and CA activity in the posterior gills is believed to be necessary in order to provide the high levels of activity needed to support the correspondingly high levels of active ion uptake across the gills (Henry, 2001).

A major difference between the Na/K-ATPase and CA is the subcellular compartmentalization of the two enzymes, but this difference is entirely consistent with the hypothesized functions of the two enzymes. The Na/K-ATPase is a large, trans-membrane protein that is localized to the basolateral membrane of the gill (Towle and Kays, 1986; Towle and Holleland, 1987; Towle et al., 2001). It is believed to generate the electrochemical gradient for global trans-epithelial ion uptake across the crustacean gill (see above) through the active transport of Na^+^ from the branchial intracellular compartment to the hemolymph. CA, on the other hand, is found in multiple subcellular fractions of the gill, but the isoform that is believed to function in support of ion transport is a small, globular protein localized to the branchial cytoplasm (Henry, 1988a,b; Henry et al., 2003). It is this isoform that undergoes the large-scale (8–20-fold) induction in response to low salinity (Henry, 1988a; Henry et al., 2003). Cytoplasmic CA is believed to function in the intracellular hydration of respiratory CO_2_, forming H^+^ and HCO^−^_3_, which then serve as counterions for the apical transport/uptake of cations and anions (primarily Na^+^ and Cl^−^), respectively (Henry and Cameron, 1983; Henry, 1984, 1988b, 2001).

## The molecular basis of branchial ion transport

### Molecular tools have identified new transport molecules

A major advance in the understanding of crustacean branchial ion transport came with the incorporation and application of molecular techniques to the study of the expression of transport proteins in gills. This approach, pioneered by the laboratory of David Towle at the Mount Desert Island Biological Laboratory, resulted in opening two major avenues of investigation: (1) expanding the number of transport proteins identified in crustacean gills, and (2) understanding the potential regulatory mechanisms for the induction of transport protein and enzyme activity in low salinity.

Starting with mRNA from the gills of *C. maenas*, and using homology cloning, the Towle lab obtained the complete 2595 base pair nucleotide sequence for a sodium-proton antiporter, which satisfied all of the criteria for an osmoregulatory transport-protein (Towle et al., 1997). The Na/H antiporter was expressed most strongly in branchial tissue (vs. non-branchial tissue) in green crabs acclimated to 32–33 ppt salinity (with a slightly higher level of expression in the posterior gills), and injection of RNA into *Xenopus* oocytes resulted in an increase in Na^+^-dependent H^+^ excretion. In addition, the full nucleotide sequence of the α-subunit of the Na/K-ATPase was also determined, and high levels of mRNA expression in the gills were shown to be correlated with high levels of protein expression (Towle et al., 2001). A V-Type ATPase (H^+^-ATPase) was also identified from a partial nucleotide sequence in the freshwater crab, *Dilocarcinus pagei*, which is also expressed in significantly higher levels, along with the Na/K-ATPase, in the posterior gills (Weihrauch et al., 2004). Furthermore, a Na/K/2Cl co-transporter was identified from two species of crabs, *Callinectes sapidus* and *Chasmagnathus granulatus*, whose expression was also high in posterior, ion-transporting gills (Towle and Weihrauch, 2001; Luquet et al., 2005). Another useful by-product of the molecular investigations has been the identification of a housekeeping gene, arginine kinase, in *C. maenas* (Kotlyar et al., 2000).

Using homology cloning and quantitative, real-time PCR, two isoforms of CA were identified in *Callinectes sapidus* and *Carcinus maenas*, a cytoplasmic form (CAc), and a membrane-associated form (CAg) (Serrano et al., 2007; Serrano and Henry, 2008), confirming earlier biochemical studies. The CAg isoform is expressed in significantly higher levels in both anterior and posterior gills of both species acclimated to high salinity, while the CAc isoform is virtually undetectable. However, the CAc isoform undergoes a 100-fold increase in expression and is the predominant isoform expressed in posterior gills for both species acclimated to low salinity. The biochemical and molecular work have shown that active ion transport is accomplished by a suite of ion transport proteins and transport-related enzymes concentrated in the posterior gills.

### Regulation of expression of transport proteins at the transcriptional level

The second area in which the techniques and approaches of molecular biology have opened new avenues of investigation involves the potential mechanisms of regulation of gene expression, specifically in response to low salinity exposure. Biochemical data on enzyme induction originally pointed to two different mechanisms of induction: (1) rapid activation of existing enzyme, as found for the Na/K-ATPase (Towle et al., 1976; also, see above), and (2) slower synthesis of new enzyme, as found for CA (Henry and Cameron, 1982b). Initial evidence obtained from the blue crab acclimated to both 35 and 5 ppt salinity showed no change in mRNA expression levels of the α-subunit of the Na/K-ATPase, apparently supporting the initial hypothesis of activation via post-translational modification (Towle et al., 2001). However, further investigation revealed that branchial Na/K-ATPase mRNA expression was induced in crabs acclimated to low salinity. In *Chasmagnathus granulatus* acclimated to 30 ppt and transferred to 2 ppt salinity, mRNA expression of the α-subunit of the Na/K-ATPase increased 35–55-fold (Luquet et al., 2005). A similar pattern but of lesser magnitude was seen for the blue crab transferred from 35 to 15 ppt, and the green crab transferred from 32 to 15 ppt: Na/K-ATPase mRNA increased 4–5-fold in posterior gills only (Serrano et al., 2007; Serrano and Henry, 2008). The increase in mRNA expression of the Na/K-ATPase was approximately 10–15 fold in the posterior 5 gills of the shore crab, *Pachygrapsus marmoratus* (Jayasundara et al., 2007). The low salinity response was similar for other transport proteins as well; expression of the Na/K/2Cl co-transporter increased 10–20-fold in posterior gills of *C. granulatus* during the first 24 h of low salinity acclimation. In the freshwater crab, *Dilocarcinus pagei*, expression of the Na/K-ATPase was 4-fold higher in the posterior than in the anterior gills (Weihrauch et al., 2004). Only the H^+^-ATPase did not show consistent differences between anterior and posterior gills or consistently and significantly increase in expression in response to a low salinity challenge (Weihrauch et al., 2004; Luquet et al., 2005).

The pattern of mRNA expression in response to low salinity is more pronounced for CA. In the posterior gills of both *C.sapidus* and *C. maenas*, expression of the cytoplasmic isoform (CAc) increased approximately 10-fold within 6 h of low salinity transfer, and that value rose to 100-fold by 24 h post-transfer and remained at that level thereafter (Serrano et al., 2007; Serrano and Henry, 2008). There were no changes in mRNA expression in anterior gills. Since it has long been established that CA activity in anterior gills does not respond to low salinity (and in general does not change at all except during the molt cycle) (Henry and Kormanik, 1985), the anterior gills have been used as a control, “housekeeping tissue” in species in which reliable housekeeping genes have not been identified (e.g., *Callinectes sapidus*). The expression of the membrane-associated isoform (CAg) increased approximately 4–5-fold over that same time period, and this is believed to be correlated with the proliferation of basolateral membrane associated with the increase in the chloride cell population in low salinity. Taken together, these data strongly support a common mechanism of gene activation and protein synthesis as the basis for transport protein and enzyme induction in response to low salinity.

Interestingly, the available data also indicate that these transport proteins are induced in response to hypersaline stress as well. CA activity increased 2–4-fold in anterior and posterior gills of the shrimp, *Litopenaeus vannamei* (Roy et al., 2007). In *Chasmagnathus granulatus* exposed to 45 ppt salinity, mRNA expression of the Na/K-ATPase, the Na/K/2Cl co-transporter, and the H^+^-ATPase all increased in the posterior gills (Luquet et al., 2005). Similarly, mRNA expression of the Na/K-ATPase increased in *Pachygrapsus marmoratus* exposed to 45 ppt salinity, but only in a single gill pair (gill 7 out of 9), suggesting that individual gills may be specialized for hypersaline adaptation (Jayasundara et al., 2007). These different patterns of induction also suggest a complex mechanism of regulation of gene expression in response to salinity changes.

### Carbonic anhydrase is regulation by a repressor

Most of the recent studies on the regulation of branchial gene expression have involved the cytoplasmic isoform of CA (CAc), which has been shown to be the isoform most directly involved in the support of ion transport. Eyestalk ablation (ESA), which removes the major endocrine complex of crustaceans (the X-organ/sinus gland complex) alters CA activity. For both *C. sapidus* and *C. maenas* acclimated to high salinity (35 and 32 ppt, respectively), in which they are osmoconformers and CA activity is at low, baseline levels, ESA results in a 1.5–3.5-fold increase in CA activity over 24 h to 7 days (Henry and Borst, 2006; Henry and Campoverde, 2006). This increase occurred only in the posterior gills, and it occurred in the absence of any low salinity exposure. ESA also enhanced low salinity-stimulated induction of CA activity in both species. When transferred from 33 to 11 ppt, CA activity doubled in intact green crabs after 4 days; ESA resulted in a 40% increase in CA induction over the value seen for intact crabs (Henry and Campoverde, 2006). In blue crabs transferred from 35 to 15 ppt, there was an approximate 8-fold increase in branchial CA activity in posterior gills 4 days post-transfer and this value approximately doubled as a result of ESA (Henry and Borst, 2006). Furthermore, for ESA-treated green crabs at 32 ppt, the increase in CA activity was completely abolished by daily injections of eyestalk homogenates (Henry, 2006). These initial data suggest the presence of a CA repressor localized to the eyestalk. This compound keeps CA activity at low, baseline levels in crabs acclimated to high salinities at which they are osmotic and ionic conformers and at which there is no need for high levels of activity. Upon low salinity exposure, the effects of this putative repressor are removed, and CA induction occurs. This mechanism appears to exist only in osmoregulating species: when the stenohaline, osmoconforming crab, *Cancer irroratus*, acclimated to 32 ppt, was treated with ESA, there was no increase in CA activity (Henry and Campoverde, 2006). Furthermore, when *C. irroratus* was transferred to 18 ppt (just above its lower lethal salinity limit) there was no induction of CA activity in either intact crabs or ESA-treated crabs. It is not known whether other transport-related enzymes or transport proteins are affected the same way by ESA.

## Branchial ammonia excretion

Ammonia (in this review NH_3_ refers to gaseous ammonia, NH^+^_4_ to ammonium ions, and ammonia the sum of both) is the natural waste product of protein metabolism. In addition to the deamination of suitable amino acids, a second pathway of ammonia generation, which is more effective in invertebrates than in vertebrates, occurs within the so called “purine nucleotide cycle” (Lowenstein and Tornheim, 1971; Lowenstein, 1972; Campbell, 1991). NH_3_ is a weak base with a pKa of 9.2–9.8 (depending on the ambient temperature and salinity of the media) and occurs in solutions in a pH-dependent equilibrium of uncharged, membrane permeable NH_3_ and ionic NH^+^_4_ (Cameron and Heisler, 1983). In body fluids with a physiological pH ranging roughly from 7.2 to 7.8, ca. 95–99% of total ammonia occurs in the hydrated form NH^+^_4_. Both forms of ammonia, NH_3_ and NH^+^_4_, have toxic effects, for instance, by potentially disturbing the cytosolic and/or intraorganelle pH (O'Donnell, 1997). In general, gaseous NH_3_, as a small uncharged molecule, can potentially diffuse to a certain extent across any membrane down its partial pressure gradient, either via membrane diffusion or via Rhesus proteins, recently identified as NH_3_ channels (Gruswitz et al., 2010). The charged form of ammonia, NH^+^_4_, cannot readily cross cell membranes by diffusion and requires transporters. Hydrated NH^+^_4_ and K^+^ ions have the same ionic radius of 1.45Å (Knepper et al., 1989; Weiner and Hamm, 2007), and due to their K^+^-like behavior, ammonium ions can compete with K^+^ ions for K^+^ transporting proteins such as K^+^ channels (Choe et al., 2000), the bumetanide sensitive Na^+^/K^+^/2Cl^−^ cotransporter (NKCC) (Good et al., 1984; Kinne et al., 1986) and the Na^+^/K^+^-ATPase (Skou, 1960). Accordingly, NH^+^_4_ has been shown to affect the membrane potential, for example in the giant axon of *Loligo pealei* (Binstock and Lecar, 1969), and also in mammalian neurons (Cooper and Plum, 1987).

### High levels of ammonia are toxic in a wide range of animal systems

While most studies on ammonia toxicity have investigated mammalian systems, where severe detrimental effects to the central nervous system have been described (Cooper and Plum, 1987; Marcaida et al., 1992; Knecht et al., 1997; Norenberg et al., 1997; Chan et al., 2000; Butterworth, 2002), aquatic crustaceans have also been shown to be affected by this molecule. The LC50 after 96 h of exposure to high environmental ammonia (HEA) was determined in the Sao Paulo shrimp, *Penaeus paulensis*, at 19 μmol.l^−1^ NH_3_ and 0.307 mmol.l^−1^ total ammonia (Ostrensky et al., 1992), in the crayfish, *Orconectes nais*, at 186 μmol.l^−1^ NH_3_ (Hazel et al., 1982) and in the red tail prawn, *Penaeus penicillatus*, at 58 μmol.l^−1^ NH_3_ and 1.39 mmol.l^−1^ total ammonia (Chen and Lin, 1992).

In the lobster, *Homarus americanus*, and the crayfish, *Pacifastacus leniusculus*, elevated environmental ammonia levels interfere with ionoregulatory functions (Young-Lai et al., 1991; Harris et al., 2001) and resulted in an increase in branchial ion permeability in the green shore crab, *Carcinus maenas* (Spargaaren, 1990). A recent study on marine Dungeness crabs, *Metacarciuns magister*, showed that long-term (14 day) exposure to 1 mmol l^−1^ NH_4_Cl caused a complete loss of the gill's capacity to actively excrete ammonia. In addition, a down-regulation of branchial mRNA expression levels of the Na^+^/K^+^-ATPase, H^+^-ATPase, cation/H^+^-exchanger and Rh-like ammonia transporter was observed while hemolymph ammonia levels increased to nearly 1 mmol l^−1^ (Martin et al., 2011).

Beside the effects that interfere with branchial ion transport capacities, ammonia also affects the immune system in crustaceans. In the shrimp, *Penaeus stylirostris*, environmental ammonia caused a reduction in the total number of immune active haemocytes by ca. 50% (Le Moullac and Haffner, 2000).

### Nitrogen excretion depends on the environment

Although crustaceans are sensitive to ammonia, the availability of water in aquatic species allows for the dilution of hemolymph ammonia to non-toxic levels, usually between 50 and 400 μmol.l^−1^ (Weihrauch et al., 2004), which is then excreted as their primarily nitrogenous waste product. Urea excretion can be considered as minor, accounting for not more than 20% of the total N-excretion in fresh- and seawater species (Delaunay, 1931; Dresel and Moyle, 1950; Needham, 1957; Jawed, 1969; Krishnamoorthy and Srihari, 1973; Weihrauch et al., 1999a,b). The main sites of ammonia excretion are usually organs also involved in osmoregulatory ion transport and gas exchange such as gills, pleopods, and other well ventilated appendages (O'Donnell and Wright, 1995; O'Donnell, 1997). In terrestrial species, the antennal gland might also play a key role in ammonia excretion, since very high ammonia concentrations (= 100 mmol L^−1^) were found, for instance, in the urine of the semi-terrestrial ghost crab *Ocypode quadrata*. The gill's function in this crab is to alkalinize the urine that passes over the epithelium. The shift in pH causes a partial volatilization of NH^+^_4_ to NH_3_, which can then be excreted as a gas (DeVries and Wolcott, 1993; DeVries et al., 1994). For a detailed review on ammonia excretion in terrestrial crabs, please refer to Weihrauch et al. (2004).

### The mechanisms of branchial ammonia excretion

The mode of branchial ammonia excretion in crustaceans is not fully understood to date. Traditionally, ammonia excretion in aquatic invertebrates was thought to be primarily a passive process (Baldwin, 1947; Kormanik and Cameron, 1981). However, recent evidence has shown that ammonia is also excreted via transport molecules, often in an active mode, as shown for the marine Dungeness crab, *Metacarcinus magister*, and edible crab, *Cancer pagurus*, brackish water green shore crab, *Carcinus maenas*, and freshwater acclimated Chinese mitten crab, *Eriocheir sinensis* (Lucu et al., 1989; Weihrauch et al., 1998, 1999a,b, 2002; Martin et al., 2011).

### Ammonia excretion across crustacean gills is dependent on transport enzymes

#### The Na/K-ATPase

The most important molecular transport component in branchial ammonia excretion in aquatic crustaceans is the basolaterally localized Na^+^/K^+^-ATPase (Riestenpatt et al., 1996; Towle et al., 2001; Tsai and Lin, 2007). Shortly after the discovery of the “Na^+^-pump” in a study on leg nerves prepared from green shore crabs, *C. maenas*, Jens Skou showed that this pump also accepts NH^+^_4_ in addition to K^+^ ions as a substrate (Skou, 1960), a finding also confirmed by investigations employing membrane vesicles from branchial epithelia of the blue crab, *Callinectes sapidus* (Towle and Holleland, 1987). Most interestingly, Masui et al. (2002, 2009) were able to show that the branchial Na^+^/K^+^-ATPase from the marine swimming crab, *Callinectes danae*, is synergistically stimulated by NH^+^_4_ and K^+^ ions. Catalytic activity was enhanced by up to 90% when NH^+^_4_ ions were added to the assay which had been previously optimized for running with K^+^ ions alone. It was speculated that the two ions bind to different sites of the Na^+^/K^+^-ATPase. A similar finding was made for the branchial Na^+^/K^+^-ATPase of the freshwater shrimp, *Macrobrachium olfersii*, by Furriel et al. (2004). For this species it was suggested that at high NH^+^_4_ concentrations the enzyme exposes a new binding site for the NH^+^_4_ ion which, after binding to NH^+^_4_, modulates the activity of the pump independently of K^+^ ions.

Also, transport studies employing the preparation of the isolated perfused gill revealed the importance of the Na^+^/K^+^-ATPase in ammonia excretion. In the marine crab, *Cancer pagurus*, active branchial ammonia excretion was completely inhibited by ouabain (Weihrauch et al., 1999a,b), whereas in the gills of brackish water acclimated crabs (*C. maenas*), active ammonia excretion was only partially (ca. 60% of controls) inhibited by the drug Weihrauch et al. (1998).

Although the Na^+^/K^+^-ATPase plays a major role in branchial ammonia excretion, a direct coupling to osmoregulatory NaCl uptake, as suggested by Spargaaren (1982) is unlikely. Gills from osmoregulatory inactive *C. pagurus* and *M. magister* crabs showed similar or even greater active ammonia excretion rates, when compared to osmoregulatory active gills from brackish water-acclimated *C. maenas* and freshwater acclimated *E. sinensis* crabs (Weihrauch et al., 1999a,b; Martin et al., 2011). Moreover, in *C. maenas*, higher ammonia excretion rates were recorded in anterior, respiratory gills, when compared to the osmoregulatory active posterior gills (Weihrauch et al., 1998).

#### The V-type H^+^-ATPase

In addition to the Na^+^/K^+^-ATPase, a V-type H^+^-ATPase (V-ATPase) also plays a major role in branchial ammonia excretion. When the marine crab, *M. magister*, was subjected to a high external ammonia concentration, gene expression of the branchial V-ATPase (B-subunit) was up-regulated (Martin et al., 2011) in order to maintain outward ammonia excretion. Further, in brackish water acclimated *C. maenas*, application of the V-ATPase inhibitor bafilomycin A1 reduced the branchial ammonia excretion rate by 66% (Weihrauch et al., 2002). Since this pump is localized to intracellular vesicles in marine and intertidal crabs (as opposed to the apical membrane in some species—see below) (Tsai and Lin, 2007; Weihrauch et al., 2001), a vesicular ammonia-trapping mechanism of excretion was suggested. It is believed that ammonia is trapped in its ionic form in acidified vesicles, which are transported along the microtubule network to the target membrane (see also Figure 9). This suggestion is supported by complete inhibition of active branchial ammonia excretion when blockers of the microtubule network, such as colchicine, thiabendazole and taxol, were applied (Weihrauch et al., 2002).

Many freshwater species of decapod crustaceans have a V-ATPase that is localized to the apical membrane of their osmoregulatory gill epithelia (e.g., *Dilocarcinus pagei* and a number of grapsid crabs: *Eriocheir sinensis, Hemigrapsus sanguineus*, and *Perisesarma bidens*) (Onken and Putzenlechner, 1995; Onken and McNamara, 2002; Tsai and Lin, 2007). Although no direct evidence has been provided to date, this enzyme is believed to be capable of functioning in an ammonia trapping mechanism that takes place across the apical cell membrane. As described for other freshwater dwelling animals such as trout and planarians, an apically localized V-ATPase acidifies the external unstirred gill/epidermal boundary layer and thereby creates a partial pressure gradient for NH_3_ (Δ P_NH3_), driving the uncharged molecule across the membrane via an apical Rh-like ammonia transporter (Rh-proteins) into the environment (Weihrauch et al., 2009, 2012; Wright and Wood, 2009).

### Ammonia transport proteins

#### The Rh proteins

**Figure 7:**
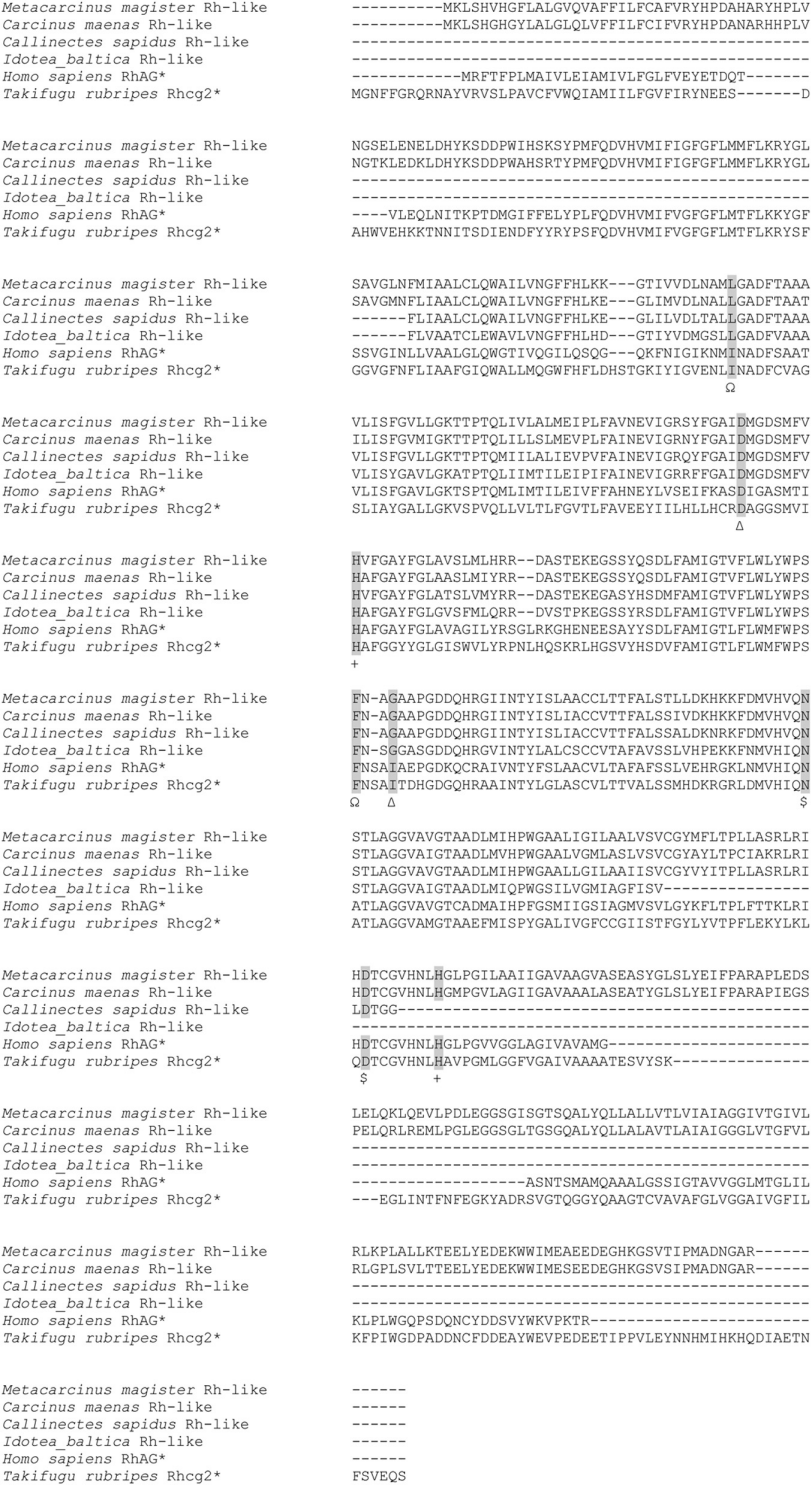
**Clustal W amino acid alignment (Thompson et al., 1994) of crustacean Rh- protein with two vertebrate Rh-proteins of verified ammonia transport capability.** Conserved ammonia-conducting residues are highlighted with a shaded background. Symbol Δ designates ammonia-conducting residues in the external vestibule; symbol Ω designates ammonia-conducting residues in the pore entrance; symbol + designates ammonia-conducting residues in the pore center; symbol $ designates ammonia-conducting residues in the internal vestibule (Khademi et al., 2004; Zidi-Yahiaoui et al., 2009; Wu et al., 2010). GenBank accession numbers are given in brackets behind the species name. *Callinectes sapidus* (AAM21147), *Carcinus maenas* (AAK50057), *Homo sapiens RhAG* (NP_000315), *Idotea baltica* (AY094181), *Metacarcinus magister* (AEA41159), and *Takifugu rubripes Rhcg2* (AB218982). Asterisks indicate Rh-proteins with verified ammonia transport capabilities (Marini et al., 2000; Nawata et al., 2010).

**Figure 8:**
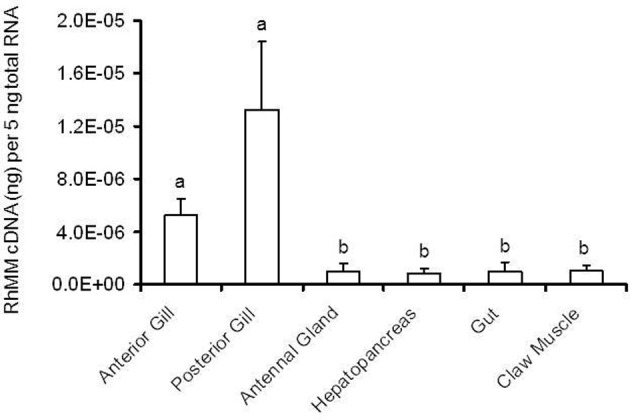
**Quantitative mRNA expression analysis of RhMM in various tissues of *Metacarcinus magister*.** Significant differences of RhMM expression are indicated by different letters (a, b). Data were analyzed employing a One-Way Anova using a Tukey's pair wise comparison. Data represent means ± SEM (*N* = 3–5).

Published so far only for mammals and fish, Rh-proteins have been shown to function as NH_3_ channels (Marini et al., 2000; Ludewig, 2004; Mak et al., 2006; Nakada et al., 2007; Nawata et al., 2010). As in many other invertebrates, the presence and expression of Rh-proteins were verified in crustacean gills (Weihrauch et al., 2004; Martin et al., 2011) and gill-like structures, e.g., pleopods of isopods (Genbank accession nr.: AY094181). A phylogenetic analysis grouped the Rh-protein from crustaceans outside the main cluster of vertebrate isoforms Rhag, Rhbg, Rhcg, and into the more primitive cluster of Rhesus-related proteins, RhP1 (Huang and Peng, 2005). Full sequence analysis from Rh-proteins cloned from *C. maenas* and *M. magister* revealed that the ammonia transporters are highly conserved proteins, showing ca. 40% identity with the vertebrate Rh-proteins with regard to the amino acid sequence, and very high similarities in the predicted secondary structures (Weihrauch et al., 2004; Martin et al., 2011). Moreover, a sequence alignment showed that critical amino acids for the NH_3_ passage are highly conserved, strongly suggesting that crustacean Rh-proteins also function as NH_3_ channels (Figure 7). Particularly high mRNA expression levels of the Rh-protein in the gill tissues of *M. magister* do support a participation of the Rh-proteins in branchial ammonia excretion (Figure 8). Due to the lack of functional antibodies, the cellular localization of Rh-proteins in the gills, however, is not known to date.

#### The apical sodium-proton exchange protein (NHE)

Another transporter that was suggested to be involved in ammonia excretory mechanisms in crustaceans is an apical localized cation/H^+^ exchanger (NHE). Evidence for this comes primarily from the inhibitory effects seen after application of amiloride, a rather unspecific blocker for Na^+^ channels, which at higher dosages also has an inhibitory effect on NHEs (Benos, 1982). In the marine osmoconforming crabs *Cancer antenarius* and *Petrolisthes cinctipes* (Hunter and Kirschner, 1986), and the green shore crab *C. maenas* (Lucu et al., 1989; Weihrauch et al., 1998) amiloride significantly reduced the ammonia excretion rates. Molecular techniques verified the mRNA expression of an Na^+^/cation exchanger in crustacean gills (Towle et al., 1997; Martin et al., 2011) that contains a characteristic amiloride binding motif (F·E·X·X·X·L·P·P·I) (Counillon et al., 1993, 1997).

However, caution should be used when interpreting results from amiloride inhibitor experiments. Studies on the isolated cuticle from *C. maenas* have shown that cuticular conductance for Na^+^ and NH^+^_4_ is inhibited by amiloride in a dose-dependent manner, with an inhibitor constant *K*amiNa^+^ = 0.6·μmol·L^−1^ for sodium ions and *K*amiNH^+^_4_ = 20.4·μmol·L^−1^ for ammonium ions, respectively (Onken and Riestenpatt, 2002; Weihrauch et al., 2002). Also basolateral potassium channels have been reported to be involved in branchial ammonia excretion (Weihrauch et al., 1998). As stated above, K^+^ and NH^+^_4_ ions have very similar biophysical characteristics (Weiner and Hamm, 2007), and NH^+^_4_ potentially substitutes for K^+^ at the K^+^-binding site of K^+^-channels. As summarized by Choe and others, the relative conductance of K^+^ channels for ammonium ions is ca. 10–20% compared to the conductance of K^+^ (Choe et al., 2000). Indeed, reduction of the branchial ammonia excretion rates in *C. maenas* after competitive inhibition of K^+^ channels by basolateral applied Cs^+^ ions, implied the participation of these cation channels in the ammonia excretion process. It is noteworthy that apical application of Cs^+^ ions had only minor effects on the ammonia excretion rates (Weihrauch et al., 1998), although K^+^- channels were shown to be present in the apical membrane of the gill epithelium (Riestenpatt et al., 1996).

### The mechanism of ammonia transport across crustacean gills

**Figure 9:**
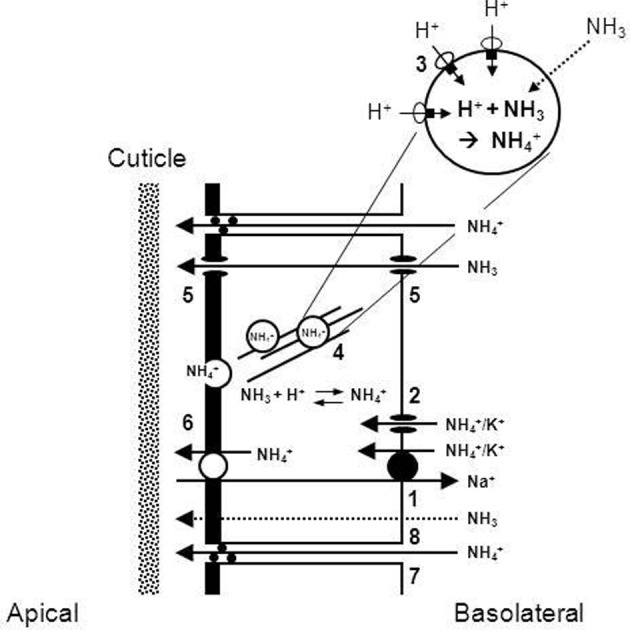
**Hypothetical model of branchial ammonia excretion in the gills of the shore crab *Carcinus maenas*.** NH^+^_4_ ions are pumped across the basolateral membrane by Na^+^/K^+^-ATPase (1) or traverse the membrane *via* potassium channels (2). Dissociation of cytosolic NH^+^_4_ to H^+^ and NH_3_ is accompanied by diffusion of NH_3_ down a partial pressure gradient into vesicles acidified by a H^+^-ATPase (3). Vesicles loaded with ammonium ions are then transported *via* the microtubule network (4) to the apical membrane where NH^+^_4_ is released into the subcuticular space by exocytosis. The localization of the Rh-protein (5) cloned from *C. maenas* gills (RhCM, GenBank Accession number: AF364404) is uncertain. Participation of an apical NHE (6), paracellular diffusion of NH^+^_4_ (7), and non-ionic transcellular diffusion of NH_3_ (8) cannot be excluded but are likely low, as shown in a transport kinetic study (Weihrauch et al., 1998). (Modified after Weihrauch et al., 2002).

With the available evidence, a hypothetical model of ammonia transport can be constructed for the gills of the green shore crab *C. maenas*, with a remaining question mark regarding the cellular localization of and overall role of the Rh-protein, cloned from the branchial tissue. The mechanism is summarized in Figure 9.

Membrane and or paracellular diffusion of ammonia across the gill epithelium cannot be excluded. Additionally, the extent of which NH_3_ diffuses through Rh-proteins in the branchial epithelium is not known to date. Recently, an aquaporin-like transporter has also been identified as being expressed in the gills of the green crab, *C. maenas* (Towle and Smith, 2006). For four members of the mammalian aquaporin family, namely AQP3, AQP7, AQP8, and AQP9, ammonia (NH_3_) transport capacities have been verified (Holm et al., 2005; Litman et al., 2009). Interestingly, a preliminary study revealed that mRNA expression levels of aquaporin in anterior and posterior gills of brackish and seawater acclimated *C. maenas* crabs directly correlate with the corresponding active ammonia excretion rates (Weihrauch, unpublished), indicating indirectly a participation of this molecule in the overall ammonia excretion mechanism.

Nevertheless, experiments monitoring gradient-driven ammonia movements across gill epithelium of the same crab species strongly suggest that diffusive, non-transporter-mediated effluxes are very small when physiological gradients (25–400 μmol L^−1^ NH_4_Cl) were applied (Weihrauch et al., 1998). Moreover, the gradient driven ammonia efflux (200 μmol L^−1^ NH_4_Cl basolateral: 0 μmol L^−1^ NH_4_Cl apical, symmetrical pH of 7.8) in this crab was inhibited by 74% when the microtubule network, and therefor the vesicular ammonia excretion process, was disrupted (Weihrauch et al., 2002). Future functional expression analysis paired with detailed localization studies of putatively involved transporters and other cellular structures must provide further insights for our understanding of branchial ammonia excretion in crustaceans.

## The role of the gill in regulating hemolymph acid-base regulation

Respiratory, ion regulatory, and acid-base physiology are all linked at the site of the gill. There are multiple causes of acid-base disturbances in crustaceans, and these depend on the environment and life history of the individual species. These sources include hypoxia/hyperoxia, hypercapnia, emersion, salinity, temperature, and exercise. A decrease in hemolymph pH is termed an acidosis, and an increase is an alkalosis. An acid-base disturbance can be of respiratory or metabolic origin. Respiratory disturbances can be illustrated by the equation below:

$${\text{CO}}_{2}+{\text{H}}_{2}\text{O}\leftarrow \text{​-----​}\to {\text{H}}_{2}{\text{CO}}_{3}\leftarrow \text{​---​}\to {\text{H}}^{+}+{\text{HCO}}_{3}^{-}$$

Respiratory CO_2_, the primary waste product of carbohydrate metabolism in cells, diffuses into the hemolymph where is it hydrated to form carbonic acid. Since the pK of the CO_2_ system is approximately 6.2 (the pH at which CO_2_ and HCO^−^_3_ are in equal concentrations) (Davenport, 1974), and crustacean hemolymph pH is typically between 7.6 and 8.0, there is nearly complete dissociation of carbonic acid to form protons and bicarbonate. If CO_2_ is produced faster than it can be eliminated, protons accumulate in the hemolymph and cause an acidosis. In air-breathing animals, extracellular fluid (blood or hemolymph) pH is controlled by changing the rate of ventilation (the rate at which the respiratory medium—air—is pumped over the surface of the gas-exchange organs – lungs and/or skin), thus regulating the rate of CO_2_ excretion.

The respiratory factors involved in acid-base regulation in aquatic crabs are different and are primarily a result of the solubility differences between O_2_ and CO_2_ in air and water. Oxygen and CO_2_ are both equally and highly soluble in air; however, while CO_2_ has equal solubility in air and water, O_2_ solubility in water is approximately 28 times lower than in air (DeJours, 1975). So while a liter of air contains approximately 200 mL of O_2_, a liter of seawater contains about 6 mL of O_2_. The consequence of this is that high volumes of water must be pumped over the gills in order to extract enough O_2_ to support metabolism. As a result of the need to maintain aerobic metabolism in a relatively O_2_-poor environment, ventilatory rate is set primarily by the levels of environmental O_2_ (Cameron, 1981). Furthermore, since CO_2_ has such a higher solubility than O_2_ in water, CO_2_ excretion occurs at a rate that is adequate to ensure that excretion matches production regardless of ventilatory rate. The partial pressure of CO_2_ (PCO_2_) in crustacean hemolymph is typically low but completely adequate to drive outward diffusion across the gill, and it is not adjusted to control hemolymph acid-base status. As a result, there are very few instances in which a change in ventilatory rate will result in an alteration of hemolymph acid-base status (i.e., hypoxia/hyperoxia, below). A metabolic acidosis is a result of the production of organic acids (e.g., lactic acid).

### Hypoxia/hyperoxia

The most prominent instances in which crustacean acid-base status changes in response to ventilator changes are hypoxia and hyperoxia. Environmental hypoxia and hyperoxia are both common in aquatic habitats (e.g., tidepools) on a circadian basis (Truchot, 1988). Under laboratory conditions simulating the natural changes in water PO_2_ in tidepools, *Carcinus maenas* underwent significant changes in hemolymph acid-base status. In hypoxia (20–50 torr), ventilation increased, resulting in an alkalosis of approximately 0.08 pH units. During hyperoxia, (350–500 torr) ventilation decreased, resulting in reduced CO_2_ excretion and a hemolymph acidosis of about 0.1 pH unit (Truchot, 1988). Interestingly, in severe hypoxia (<20 torr), an acidosis occurred accompanied by a 2.5-fold increase in hemolymph PCO_2_. This is believed to result from the fact that 20 torr is below the critical limit of the crab, the PO_2_ at which it costs more energetically to ventilate the gills than is repaid by the O_2_ taken up from the water, so ventilation ceases. Furthermore, artificial hyperventilation in normoxic *C. maenas* also caused a respiratory alkalosis (Burnett and Johansen, 1981). In crayfish subjected to hypoxia (50 torr), ventilatory rate increased 2.8-fold, hemolymph PCO_2_ was reduced by 50%, and a hemolymph alkalosis resulted (Wheatly and Taylor, 1981).

### Temperature

One of the central principles in poikilotherm acid-base physiology is that the pH of the extracellular fluid varies inversely with temperature (Rahn, 1967; Howell et al., 1973). Because of the effect of temperature on the ionization of the water molecule, the neutral pH of water changes inversely with temperature, and thus maintains a constant H/OH ratio. Temperature also alters the physical–chemical properties of the naturally occurring buffers in the extracellular fluids of animals, specifically the imidazole groups of histidine residues of proteins (Howell et al., 1970; Reeves, 1972, 1976). The result is that blood is maintained at a pH slightly above that of neutral water at any given temperature, but proteins maintain a constant net charge (and therefore continuity of function) as temperature changes. These studies led to the concept of constant relative alkalinity, which is believed to be universal in cold-blooded animals (Rahn et al., 1975).

This relationship holds for aquatic crustaceans as well. In *Callinectes sapidus* and *Carcinus maenas*, the change in pH with temperature was reported as −0.12 and −0.16 pH units/°C, respectively (Truchot, 1973; Cameron and Batterton, 1978b). The lower pH at higher temperature is accompanied by a reduction in the total concentration of CO_2_ in the hemolymph (primarily HCO^−^_3_). This decrease cannot be explained by passive processes alone (Cameron and Batterton, 1978b), indicating that some active mechanism of compensation is operating at the site of the gill.

### Exercise

Members of the family Portunidae (aquatic swimming crabs) are very active and undergo both short- and long-term bouts of exercise in nature. Exercise has been shown to produce a mixed respiratory/metabolic acidosis in crabs (Booth et al., 1984). When *Callinectes sapidus* were induced to swim for a period of 1 h, hemolymph PCO_2_ doubled within 15 min and remained high throughout the exercise period, pH was depressed by 0.4 units at 15 min and remained low, and lactate increased continuously throughout the exercise period (by 5 mM at 30 min and 9 mM at 60 min) (Booth et al., 1984). The interesting aspect of these results that shed light on the role of the gill in this process is that the amount of lactate measured in the hemolymph during exercise was much greater than H^+^, despite their being produced in a 1:1 ratio. This discrepancy was also seen in the hemolymph of another species, *Cancer magister*, during exercise (McDonald et al., 1979), and it was originally postulated that protons were sequestered in muscle tissue while lactate was released. However, the study on *C. sapidus* also included measurements of acid excretion into the surrounding seawater, and the results showed that enough acid was excreted to fully account for the difference in the hemolymph. This could have occurred either via direct H^+^ excretion or as NH^+^_4_ excretion, as the latter also increased by 6-fold during exercise (Booth et al., 1984). Regardless, these results clearly indicate that the gill is a major site of hemolymph pH compensation during and after exercise. It was suggested that H^+^ (or NH^+^_4_) excretion could occur in exchange for the uptake of Na^+^, which would preserve the electrical neutrality of the hemolymph.

### Salinity

In the few studies on crustacean acid-base balance and environmental salinity, it has been reported that hemolymph pH varies inversely with salinity. Mangum et al. (1976) were the first to report this for the blue crab, *Calllinectes sapidus*, and their initial conclusions were that excess hemolymph ammonia (a weak base) in crabs acclimated to low salinity contributed to the increase in pH. In further studies on the blue crab, it was found that low salinity acclimation resulted in a metabolic alkalosis: increased pH and increased HCO^−^_3_ at constant PCO_2_ (Henry and Cameron, 1982b). A similar pattern was also seen in *Carcinus maenas* (Truchot, 1981) and *Eriocheir sinensis* (Truchot, 1992). In blue crabs, there was also a change in the difference between the two major hemolymph ions, Na^+^ and Cl^−^: both decreased in low salinity but not to the same degree. For transfer from 29 to 8 ppt (865 to 250 mOsm kg H_2_O^−1^), Na^+^ decreased by 90 mM while Cl^−^ decreased by 111 mM. The difference, which was negative at 29 ppt, became positive at 8 ppt, and this was taken as a general indicator of changes in the overall strong ion difference (SID) in the hemolymph. The SID is an important factor in influencing the acid-base status of extracellular fluids (Stewart, 1978). The concentrations of strong ions, such as Na^+^ and Cl^−^, are maintained independently, while those of weak ions (H^+^, OH^−^, and HCO^−^_3_) are adjusted to maintain the electrical neutrality of the fluid compartment. This makes resting acid-base status dependent on the regulation of the ionic concentrations in the hemolymph at different salinities; and at low salinity, HCO^−^_3_ apparently increases to help offset the changes in the SID, resulting in an alkalosis. The strong ion concentrations are maintained by the branchial transport processes described above, thus linking hemolymph acid-base status with ion regulation. Conversely, a metabolic acidosis developed in the crayfish, *Pacifastacus leniusculus*, when transferred from low to high (75% seawater) salinity, and this was correlated to the onset of hypo-osmotic and ionic regulation of the hemolymph (Wheatly and McMahon, 1982).

Studies on the enzyme CA have also made the link between branchial ion transport, hemolymph ion regulation, and acid-base status more clear. Again, in blue crabs acclimated to low salinity (8 ppt), inhibition of branchial CA with acetazolamide resulted in a dose-dependent decrease in osmolality and the concentrations of Na^+^ and Cl^−^. However, Cl ^−^ concentrations declined more than Na^+^, thus increasing the SID and causing a significant metabolic alkalosis to develop (Henry and Cameron, 1983). CA inhibition by acetazolamide affects Na^+^ and Cl^−^ transport differently: the drug causes decreases in the active uptake of both ions in low salinity, but it also causes a large increasing in the outward flux of Cl^−^ (Cameron, 1979), and this could be the underlying reason for the disruption of the SID. These studies established the link between branchial ion regulation and hemolymph acid-base status, and experimental work using hypercapnia (elevated CO_2_) has suggested that adjustments in the relative rates of Na^+^ and/or Cl^−^ transport could be a mechanism for compensation of acid-base disturbances.

### Hypercapnia

Because of the high solubility of CO_2_ in water, and because over 95% of the total dissolved CO_2_ is in the form of HCO^−^_3_ ions, most aquatic crustaceans that live in large, well-mixed bodies of water rarely experience environmental hypercapnia. Aquatic hypercapnia can develop in restricted habitats such as tide pools and coastal tributaries (e.g., Truchot, 1988; Cochran and Burnett, 1996). However, experimental hypercapnia in the laboratory has been one of the most effective tools with which to study the mechanism of acid-base regulation. Cameron (1978b) presented the first experimental evidence to support the original hypothesis of Krogh (1938), that acid-base regulation and branchial ion transport could be linked through processes of Na^+^/H^+^ or Na^+^/NH^+^_4_ exchange and Cl^−^/OH^−^ or Cl^−^/HCO^−^_3_ exchange. Blue crabs exposed to 1% CO_2_ in water (7.5 torr) rapidly developed a respiratory acidosis of 0.3 pH units. pH then rose over next 48 h, indicating a partial compensation of non-respiratory origin at constant PCO_2_, as there were no changes in ventilator rate. Na^+^ and Cl^−^ fluxes were measured isotopically. Despite measuring relatively small changes against a high background, a measurable increase in the ratio of Na^+^ influx to Cl^−^ influx was found. These data strongly suggest that there was a higher rate of Na^+^/H^+^ exchange, and hence, more H^+^ excretion as a result of the hypercapnic acidosis. Despite this work being performed 34 years ago, it is still the best direct evidence that ion transport processes also function in the regulation of hemolymph acid-base status and can be adjusted to compensate for an acid-base disturbance. In aquatic marine crustaceans, the contributions of both the antennal gland (to ion transport) and the shell (to buffering via CaCO_3_ dissolution) have been shown to be minimal, and that transport by the gill is the primary mechanism of acid-base regulation (Cameron and Batterton, 1978a; Cameron, 1985).

## Branchial transport and bioaccumulation of toxic metals

### Examples of toxic metals

Estuarine and coastal aquatic environments are known sinks for the accumulation of toxic metals, resulting from both natural phenomena (e.g., volcanic activity and soil erosion) and anthropogenic sources (e.g., industrial and agricultural effluents). Metals are particularly harmful to living systems because (1) they are non-degradable, and (2) they tend to accumulate within the organism. The biological interactions of metals (i.e., from the d-block category of the periodic table) are based on the fact that these metals have an extremely wide range of redox potentials and complex formations (Duffus, 2002). From this group we will report on the transport and bioaccumulation of Cu^2+^, Zn^2+^, Cd^2+^, Hg^2+^, and Ag^+^ in aquatic Crustacea. Within this group, the essential metals Cu^2+^ and Zn^2+^ participate in redox reactions controlling transport of electrons and participating as ligands in complex enzymatic reactions in biological systems. However, if the concentrations of these bioelements are too high at the source of supply, the homeostatic mechanism ceases to function and they act acutely or chronically as toxic metals. We will also include one of the d-block metals with a higher atomic number, Pb^4+^/Pb^2+^. The biological roles of the non-essential metals Cd^2+^, Hg^2+^, Pb^2+^, and Ag^+^ have not yet been established, but they could potentially substitute for an essential metal of lower affinity and distort the geometry of the natural molecules, resulting in numerous morphological and physiological disfunctions.

### How toxic metals are accumulated in crustaceans

Toxic metals are taken up and accumulate in aquatic Crustacea directly from their environment across the gills or other body surfaces, and from their diet via the intestine. Unlike marine teleosts, marine and brackish water crustaceans do not drink seawater (the exception being *Artemia* when acclimated to a hyperosmotic medium—Smith, 1969). During waterborne exposure to toxic metals, crustacean gills, which are the major entry site, act mostly as a transient tissue store for accumulated metals (Soegianto et al., 1999). Gills provide a selective interface between the external environment and the internal milieu, and as such they are in permanent danger of accumulating toxic substances from a polluted environment. In the branchial cavity the same large lamellar surface area, high permeability, and countercurrent flow of water and hemolymph that facilitate the exchange of respiratory gasses and inorganic osmolytes also enhance the uptake of toxic metals (Foster and Howse, 1978). Waterborne toxic metals interact with crustacean gills by adsorption to the cuticular side, by transport across the epithelium cells, and by trafficking into the hemolymph and subsequent bulk transport to internal organs.

Chitinous building blocks of the cuticle are very efficient in binding trace metals. A type of pore canal is described from the cuticle of *Gammarus* (Muzzarelli and Tuberni, 1969). Each canal passes from the epidermis vertically through the endocuticle and exocuticle, and in the most distal layers of the latter is slightly expanded. Each pore canal is connected to many necks in which openings are aligned in rows about 0.15–1.0 μm apart. Changes in the pore and canal contents are visible, but their significance in the transport of small molecules has not been well-studied (Halcrow, 1978). Ca^2+^ is absorbed across the microvilli of the epithelial cells, which span the entire part of cuticular matrix (Compere et al., 1993). In aquatic larvae of *Simulium ornatipes* (Diptera), cuticular zinc is bound by compounds containing phenolic groups and by phosphoric acid, which form a weak bond with the zinc atom (Carter and Nicholas, 1978). The cuticle and epicuticle of lobster gills are the main targets for binding of cadmium and zinc, and the transfer of ionic forms across the epithelium cell membrane is directed by a protein carrier-mediated process (Rainbow, 1997). Freshwater and estuarine malacostracans have a lower cuticular permeability for the major inorganic ions, and hence metals, compared with related marine species (Pequeux and Lignon, 1991).

### Waterborne exposure of crustacea and transport of toxic metals across the gills

Accumulation of toxic metals in the gills is typically studied after waterborne exposure of the whole organisms in seawater containing radioactively labeled trace metals (Bryan, 1968) or by perfusion of the gills with radioactive or stable metals (Pedersen and Bjerregaard, 1995, 2000; Lucu and Obersnel, 1996; Norum et al., 2005). When blue crabs (*Callinectes sapidus*) were exposed to waterborne cadmium, the metal rapidly crossed the gill epithelium and was transported via the hemolymph to the hepatopancreas (Brouwer and Lee, 2007). Studies on the crabs suggested that the amino acid histidine chelates the cadmium, and that the histidine-cadmium complex competes with unchelated histidine for absorption across the mucosa epithelium from the hemolymph to the hepatopancreas (Wright, 1977; Pecon and Powell, 1981; Martin and Rainbow, 1998).

The rate of cadmium influx across the gills of aquatic organisms depends on the availability of the trace metal ion concentration in seawater (Sunda et al., 1978; Engel and Fowler, 1979). Transfer of ionized cadmium across the isolated perfused *Carcinus* gill preparation was suggested to occur via a transcellular pathway with a minor role for paracellular transport (Pedersen and Bjerregaard, 2000). Cadmium enters the freshwater amphipod *Gammarus pulex*, and the brackish water *Carcinus maenas*, at least in part in competition with uptake mechanisms for Ca^2+^; the rate of Cd uptake is inversely related to the calcium concentration in the medium (Wright, 1977; Wright and Frain, 1981; Bjerregaard and Depledge, 1994; Lucu and Obersnel, 1996; Pedersen and Bjerregaard, 2000). Toxicity of waterborne cadmium may therefore lie in the perturbation of calcium metabolism. The sensitivity of brine shrimp to cadmium toxicity is decreased by the Ca^2+^ channel inhibitor diltiazem, indicating a possible role of the Ca^2+^ channel in Cd^2+^ uptake in that organism (Borowitz and McLaughlin, 1992). When perfused gills of *C. aestuarii* and *C. maenas* were exposed to the non-specific Ca^2+^ inhibitor lanthanum in the bathing solution, the influx of cadmium across the apical side of the gill is partially or completely inhibited (Lucu and Obersnel, 1996; Pedersen and Bjerregaard, 2000).

In gill cells dissociated from filaments of the lobster *Homarus americanus* and separated via sucrose gradient, uptake of ^65^Zn was measured in the presence and absence of Ca. Uptake of ^65^Zn followed a hyperbolic type of Michaelis–Menten kinetics (Sa et al., 2009). The gill cells separated in the low 30% sucrose gradient and incubated in saline with 10 mM Ca^2+^ had reduced apparent ^65^Zn affinity and maximum transport velocity compared with Zn uptake in Ca^2+^-free medium. However, in the cells separated in 80% sucrose gradient, maximum transport velocity of ^65^Zn was 2-fold greater in saline containing 10 mM Ca^2+^. Therefore, the inhibitory or stimulatory effects of calcium on ^65^Zn uptake depend on the type of cells separated in high or low sucrose density gradient. In isolated gill filaments, uptake of ^65^Zn showed similar transport properties as the cells separated in the 80% sucrose gradient, suggesting apical cell uptake of ^65^Zn. Increased Ca^2+^ concentration in the external solution and the presence of Ca^2+^ inhibitors (verapamil and fadrasole) reduced affinity and maximum transport capacity of the radioactive zinc (Sa et al., 2009).

Waterborne exposure of the shore crab, *Carcinus maenas*, to HgCl_2_ and MeHg (CH_3_HgCl; 50 μg L^−1^) *in vivo*, or by perfusion of the isolated gill epithelium, resulted in a high degree of bioaccumulation of the metals by the gill epithelium (Laporte et al., 2002). Highly lipid-soluble complexes of MeHg and Hg^2+^ in the form HgCl_2_ rapidly cross cell membrane. MeHg is more homogenously and rapidly distributed than inorganic mercury. In the medial portion of the gill stem mercury is randomly distributed within lamellae in the branchial “kidney cells”—nephrocytes. The central vacuole of the gill nephrocyte is the main storage site for HgCl_2_ and MeHg. Evidence from the distribution of the different chemical species of Hg suggests that mercury is transferred into intracellular vesicles (Lawson and Mason, 1998; Laporte et al., 2002). When the radioactively labeled inorganic form of ^203^Hg accumulates in the crayfish *Orconectes propinquus* after 14 days of dietborne exposure, the maximum concentration of mercury was found in the hepatopancreas, with half of that value in the gills. Following a 21-day period of metal efflux, about 2/3 of the mercury content of gills was depurated, but the hepatopancreas content remained stable.

Hemolymph sodium concentration was lowered in crayfish exposed to mercury (Wright and Welbourn, 1993). The unfolded epipodite (hemiepipodite- one epithelium cell layer supported by cuticle) isolated from branchial cavity of the lobster *Homarus gammarus* was mounted in an Ussing type chamber, and the effects of apically added MeHg on the short-circuit current (Isc) was studied (Lucu et al., 2009). Apical addition of MeHg depressed short-circuit current with half-maximal inhibition concentration (IC_50_) of 0.6 μ M and inhibition kinetics suggesting the presence of a saturable and cooperative carrier-mediated transport system. The concentration of 3 μ M MeHg or HgCl_2_ inhibits 98% of Isc (apical side). Basolateral application of 3 μ M HgCl_2_ has no effect on Isc. The reducing agent 1,4-dithiothreitol (DTT) partially recovered the MeHg induced block of Isc, suggesting that the formation of S-Hg-S bridges is important in the inhibitory mechanism. In the hemiepipodite preparation, apically added MeHg inhibits Na^+^ and Cl^−^ influxes (Lucu et al., 2009), which indicates inhibition of an apically located Na^+^/K^+^/2Cl^−^ cotransporter (Lucu and Towle, 2010). An Na^+^/K^+^/2Cl^−^ transporter was inhibited by MeHg in the membrane vesicles isolated from shark rectal gland (Kinne-Saffran and Kinne, 2001) and shark and human orthologs (Weber et al., 2006). Mercury alters the function of transport proteins by reacting with cysteinyl sulphydryl (SH) groups, thus affecting protein conformation changes (Zalups, 2000).

The initial effect of copper and silver on osmoregulation in the water flea, *Daphnia pulex*, was found by Holm-Jensen (1948). Comparative studies on gills of teleost fish and crustaceans showed similar entry pathways for copper and silver (Grosell et al., 2002). Na^+^ and Cu ^2+^ have similar mobilities in solution (λ for Na^+^ = 50.1 and for Ca^2+^ is 53.6 cm^2^ ohms^−1^ equiv^−1^). When *Carcinus maenas* is exposed to elevated levels of waterborne copper, the metal is taken up by the gills. Intracellular Cu^2+^ inhibits basolaterally located Na^+^ /K^+^- ATPase with the result being the decreased unidirectional sodium transport of Na^+^ from the gill into the blood and the decrease of blood Na^+^ concentration (Handy et al., 2002).

Toxic effects of Cu^2+^ were studied in the blue crab *Callinectes sapidus* acclimated to 2 ppt dilute seawater (Martins et al., 2011). Candidates for copper entry across gill epithelium include transport systems for Na^+^ and Cl^−^ (Martins et al., 2011). These include the apically localized Na^+^ /K^+^/2Cl^−^ co-transporter in the gills of hyperosmoregulating Crustacea (Riestenpatt et al., 1996), epipodite (Lucu and Towle, 2010), and a Cl^−^/HCO^−^_3_ exchanger or Na^+^ -channel transporters (Glover and Wood, 2005; Tresguerres et al., 2008). Pharmacology of the specific Cu^2+^ carrier is yet unknown either in fish or Crustacea. The hemolymph protein hemocyanin binds copper and ensures the transport of the remaining copper to tissues occurs in a less toxic form (Rtal et al., 1996; Truchot and Rtal, 1998). Cells dissociated from the posterior gills of *Callinectes sapidus* (acclimated to dilute seawater) and incubated in 50 or 100 μM copper, show a linear uptake rate of copper with a several fold higher accumulation in gill cells than in hepatopancreas cells (Paganini and Bianchini, 2009).

Whole-body uptake of waterborne silver in the neonates and adult stages of *Daphnia magna* is also closely dependent on Na^+^ transport mechanisms. In the branchial epithelium of neonates, an Na^+^ channel coupled with a V-type-H-ATPase activity is the driving force for Na^+^ uptake, and Ag^+^ competes with Na^+^ for transport via this mechanism (Bianchini and Wood, 2003).The lack of this mechanism in adults results in less efficient entry of silver by a putative Na^+^ channel associated with an electrogenic 2Na^+^/H^+^ exchanger (Shetlar and Towle, 1989). This results in a lower toxicity of Ag^+^ in adults. A negative correlation between accumulation of toxic metals and body mass was found. It was hypothesized in the neonates of the water flea *Daphnia* that Na^+^ is basolaterally transported into the hemolymph from the cell by an Na^+^/Cl^−^ cotransport; however, in adults this role is probably taken by Na^+^/K^+^/2Cl^−^ cotransport (Bianchini and Wood, 2003; Glover and Wood, 2005). Interestingly, organic matter and humic substances present in the water protect the neonates of *Daphnia magna* against copper toxicity (Al Reasi et al., 2012).

### Effects of toxic metals on gill transport-related enzymes: the Na^+^/K^+^-ATPase and carbonic anhydrase

SH groups of the Na^+^/K^+^ -ATPase are the major targets of covalent binding of toxic metals. Cadmium exerts an inhibitory effect on the Na^+^/K^+^ -ATPase from the cytoplasmic side of the cell membrane (Tokushige et al., 1984). At the molecular level, Pb^2+^ interferes with the hydrolytic cleavage of the phosphorylated intermediate E_2_P during the K^+^ translocation step of the pump (Gramigni et al., 2009). Silver competes with the magnesium binding site on the Na^+^/K^+^ -ATPase and thus has an inhibitory effect on enzyme phosphorylation (Ferguson et al., 1996). Weakening of the membrane machinery of the α- subunit of Na^+^/K^+^ -ATPase by mercury leads to its release from the membrane, preferentially in the E_2_ conformation (Imesh et al., 1992).

When the freshwater amphipod, *Gammarus fossarum*, was exposed to 15 μg/L Cd (3 and 7 day exposures), hemolymph osmolarity decreased significantly, fluorescence intensity of the immunolocalized Na^+^/K^+^ -ATPase was diminished, and damage to the cellular morphology of the gills were all evident. The Na^+^/K^+^ -ATPase α-subunit mRNA was elevated during the first three days of exposure to cadmium but afterwards was down-regulated to the control value (Issartel et al., 2010). In the gills of the blue crab *Callinectes sapidus* acclimated to 2 ppt seawater, low copper concentration inhibited expression of mRNA genes encoding the Na^+^/K^+^-ATPase. Gene transcription (i.e., mRNA expression) of the Na^+^/K^+^-ATPase was down-regulated before the enzyme was inhibited. These effects were not expressed in the gills of the blue crab acclimated to 30 ppt salinity seawater (Martins et al., 2011). Copper inhibits the gill Na^+^ K^+^-ATPase activity and decreases the hemolymph osmotic concentration in juveniles of the freshwater prawn *Macrobrachium rosenbergii*. Increase of the intracellular Cu^2+^ concentration induced competition with Mg^2+^ for the binding site of the ATP molecule, rendering the Na^+^/K^+^-ATPase unable to generate normal transport of Na^+^ into the hemolymph (Li et al., 2009).

Cadmium and copper inhibition of branchial Na^+^/K^+^- ATPase activity resulted in reduced hemolymph ion levels (Hansen et al., 1992; Postel et al., 1998). In the freshwater amphipod *Gammarus pulex* copper concentrations of 100 μg/L^−1^ caused a significant reduction in Na^+^,K^+^- ATPase activity and consequently inhibited sodium influxes and hemolymph sodium concentration (Brooks and Mills, 2003).

The mode of action of Cd^+^ on the Na^+^/K^+^- ATPase varies in different species. In the amphipod, *Gammarus pulex*, exposed for several days to cadmium (0.75 and 15 μ g/L), the activity of the Na^+^/K^+^- ATPase was significantly increased, and osmolarity and Ca^2+^ concentrations in the hemolymph decreased (Felten et al., 2008). Responses correspond to the changes of the enzyme conformation and/or in the membrane protein—lipid interaction affected by trace metals resulting in the inhibition or stimulation of the Na^+^/K^+^- ATPase activity.

In the Chinese mitten crab, *Eriocheir sinensis*, acclimated to fresh water, there was a significant decrease in the activity of the Na^+^ /K^+^ -ATPase in the anterior gills, a significant drop in osmolarity, hemolymph Na^+^ and Cl^−^ concentrations, and cytochrome c oxidase activity after acute exposure to waterborne cadmium for 1, 2, or 3 days (0.5 mg Cd/L; Silvestre et al., 2005). The ultrastructure of the anterior gills was also found to be impaired. In the posterior gills, which are mostly specialized for osmoregulation, cuticlular and cellular morphology were not changed. After acute Cd exposure, crabs were then exposed chronically (30 days) to the lower concentrations of 10 or 50 μg Cd/L. Chronic exposure to low concentrations of cadmium allowed crabs to recover both osmoregulatory capacity and Na^+^ /K^+^ -ATPase activity; anteror gill ultrastructure also appeared to recover. Recovery of the transport mechanism of the crabs after low-level cadmium exposure was interpreted as acclimation to low-level metal contamination (Silvestre et al., 2005).

Gill Na^+^/K^+^- ATPase and Ca^2+^/Mg^2+^-ATPase activities decreased in the juvenile Chinese mitten crab, *Eriocheir sinensis*, when ambient mercury (HgCl_2_) concentrations and exposure times increased (Zhao et al., 2011). Mercury had an inhibitory effect on the plasma membrane enzyme, resulting in elevated Na^+^ concentrations in the cytosol and a concomitant induction/synthesis of metallothioneins (MT). This observation led the authors to suggest that Na^+^ has a role in MT synthesis (see below) (Imesh et al., 1992).

The gill CA of the grapsid crab *Chasmagnathus granulata* was inhibited *in vivo* by cadmium (IC_50_ = 1.6 mg/L), but only in extremelly diluted seawater (2.5 ppt) was there a significant reduction in the concentration of Na^+^ in the hemolymph. Under *in vitro* condition IC_50_ of CA for Cd^2+^ and Cu^2+^ was 0.022 mM and 0.038 μM, respectively, and for Zn^2+^ 3.8 μM (Vitale et al., 1999). Inhibition of CA by toxic metals restricts the role of the enzyme in providing acid-base equivalents needed for ion exchangers (Henry, 1996). In the species *Carcinus maenas*, the cytoplasmic CA isoform is more resisent to toxic metal pollution than is the same CA isoform from the blue crab *Callinectes sapidus* (Skaggs et al., 2001; Skaggs and Henry, 2002). For cytoplasmic CA from gills of the blue crab, the inhibition constants (Ki) for Ag^+^, Cd^2+^, and Cu^2+^ are in the range from 50 to 500 nM. CA isolated from microsomal fraction located at the basolateral membrane side of the gill epithelium is less sensitive to metals than the cytoplasmic fraction. It was suggested that in the microsomal fraction metals might be sequestered in the lipid component; therefore lower amount of metals in this fraction are available for CA inhibition. The larger difference in K_i_ values suggests the presence of species-specific CA isoforms in crustaceans.

### Metallothionein like proteins and metal containing granules

Metals are detoxified through their binding to metallothionein-like proteins (MTLP), accumulation in intracellular vacuolar granules, or through the formation of extracellular granules. MT are metal-thiolate polypeptides present in plants and animals characterized by low molecular mass and rich in cysteine. Their properties include the ability to bind metals and resistance to heat. MT from marine invertebrates appear to differ from the vertebrate MT by their lower cysteine content and sometimes by the presence of aromatic residues. In crabs there is a class of proteinaceous MTLP with cysteine concentrations close to those of mammals (Lerch et al., 1982; Brouwer et al., 1989; Binz and Kãgi, 1999). The crustacean amino acid sequence of the two MTLP isoforms were found in the mangrove crab *Scylla serrat*a (Lerch et al., 1982), which is similar to that of MTLP from *Homarus americanus* (Brouwer and Brouwer-Hoexum, 1991), *Carcinus maenas* (Pedersen et al., 1994), *Callinectes sapidus* (Brouwer et al., 1995), and the freshwater crayfish *Astacus astacus* (Pedersen et al., 1996). MTLP isoforms, which differ in primary sequence, are MT-10 and MT-20. In Crustacea, the transcription of MT-20 genes and MT-10 genes was induced by cadmium (Pedersen et al., 1998). Cadmium in the gill and hepatopancreas is bound to the low-molecular weight metal-binding MTLP. Two CdMT isoforms are present in the hepatopancreas, but only one in the gills (Brouwer et al., 1984). For the blue crab, *Callinectes sapidus*, three metallothionein encoding genes were identified, suggesting that a specific metallothionein is involved in copper homeostasis associated with synthesis and degradation of hemocyanin (Brouwer et al., 2002). Concentrations of the Cu-thionein in crustaceans depend on the hemocyanin availability for oxygen transport, suggesting its involvement in the regulation of hemocyanin activity. In the American lobster, *Homarus americanus*, hepatopancreas metallothionein-cDNA studies revealed the presence of zinc thionein as a protein that binds zinc *in vivo* rather than copper (Valls et al., 2001).

Increased turnover of MTLP occurs in response to elevated metal concentrations (Hogstrand and Haux, 1991; Mouneyrac et al., 2002). The correlation between MT levels in tissues and the environmental concentrations of toxic metals has led many authors to suggest that MTs are useful as biomarkers of environmental contamination. The markedly elevated level of Zn^2+^ in the gills of crabs living in polluted waters (Mouneyrac et al., 2001) displayed a seasonally-dependent pattern that correlated with environmental zinc availability. The subcellular, heat-stable fraction of metals associated with a 7.5 kDa MTLP in the cytosol was induced by the presence of toxic metals in the environment. In the redox reaction, Zn^2+^ may be displaced from metallothionein by Cd^2+^ or other metals (Canli et al., 1997; Engel, 1991; Amiard et al., 2006).

Induction of MTLPs by elevated concentrations of Cd, Cu, and Zn was analysed in gills and hepatopancreas of the crabs *Pachygrapsus marmoratus* and *Carcinus maenas* in the metal contaminated area of the river Gironde (Legras et al., 2000). In the European shore crab, *Carcinus maenas*, induction of MTLP was correlated with the environmental concentrations of metals (Pedersen et al., 1994; Legras et al., 2000). Induction of MTLPs in the crab *Pachygrapsus marmoatus* was correlated with crab metabolism (i.e., changes in total body protein concentrations) under different salinity conditions rather than with the elevated metal concentrations (Legras et al., 2000). Changes in the concentrations of MTs in *Pachygrapsus marmoratus*, induced by low salinity, depended on the chemical species and bioavailability of metals in the environment. In addition to metal concentrations, abiotic factors such as salinity and temperature, and biotic factors such as period of reproduction and moulting, may affect induction of the MTLP (Mouneyrac et al., 2001). For example, cytosolic MTLP concentrations in posterior gills were 2.6-fold higher in *Carcinus aeastuarii* acclimated for more than two weeks in 12 ppt salinity and taken from non-poluted areas than in gills of crabs taken from 38 ppt salinity seawater (Lucu et al., 2008). MTLP concentration significantly increased in posterior and anterior gills at 2 and 24 h, respectively, after hypo-osmotic stress. MTLP was also increased under *in vitro* conditions when anterior or posterior gills were perfused with hemolymph isosmotic saline and incubated in environmental hypo-osmotic saline. In the posterior gills perfused with isosmotic saline, MTLP concentrations in gills were inversely correlated with the calcium concentration in the external bath (De Martinez et al., 2009). Taking into consideration the multifunctional roles and causes of induction of MTLP by both toxic metals and biotic and abiotic factors, we have to take with caution the role of crustacean branchial MTLPs as environmental biomarkers for toxic metals.

### Toxic metals and reactive oxygen species

Toxic metal pollutants cause stress under natural and artificial conditions by disturbing the balance between the production and elimination of reactive oxygen species (ROS). The role of MTs in the antioxidant defense is affected by both toxic metals and by other environmentally induced changes. Hyposmotic stress generates an increase in the ROS followed by lipid peroxidation, and this also causes induction of MTLP (Viarengo et al., 2000).

In the blue crab *Callinectes sapidus*, exposed to hyposmotic stress for 24 h, high activity of mitochondrial cytochrome oxidase in the gills (Welcomme and Devos, 1988) contributes to the antioxidant defense and alleviation of the oxidative stress response (De Martinez et al., 2009). During the summer season, when the crustacean *Scylla serrata* was subjected to salinity and temperature fluctuations, increases of the activities of gill transport proteins (Na^+^,K^+^- ATPase, Mg^2^-ATPase, Mg^2+^,Ca^2+^ -ATPase and Ca^2+^ -ATPase), MTLP, and antioxidant enzymes (i.e., superoxide dismutase, catalase, glutathione peroxidase) were evident. The final product of the increase of ROS and lipid peroxidation is malondialdehyde (MDA). In this case MTs serve in an antioxidant role (Kong et al., 2008). In the non-polluted environment, induced stress may facilitate the reactivation of Zn^2+^-containing enzymes and chelation of Zn^2+^ to apo-MT. Mechanisms of this process remain to be studied. Zn^2+^ concentrations in the posterior gills of *C. aestuarii* induced by hyposmotic stress may be controlled by reversible complexation with newly synthesized apo-MT (Maret et al., 1999).

In addition to the actions of MTLPs, a second mechanism of cellular sequestration and detoxification of metals is found in the lysosomal system, through the formation of intracellular granules or granules that are precipitated extracellularly. Metal containing granules in isopods include bodies classified as type A, containing layers of Ca^2+^ and Mg^2+^ phosphates, which may also contain zinc. Type B bodies originate from the lysosomal system and contain mostly acid phosphatase; these accumulate sulphur, Cd^2+^, Cu^2+^, and Zn^2+^. These granules were mostly found in the hepatopancreas (Hopkin, 1989). During crustacean moulting, physiological processes that involve the binding and transport of metals have been associated with increased deposition of calcium needed for the formation of the new skeleton rather than serving as a detoxification role (Khan et al., 2012). In fish gills, Zn^2+^ and Ca^2+^ -binding intracellular proteins are mostly sequestered by endo-plasmatic reticulum, and cytosolic activities of the free ions are at a minimum (Wood, 2012).

In the shrimp, *Penaeus indicus*, Zn^2+^ is slightly more concentrated in the soluble fraction of the gills, where it is mostly deposited in nephrocytes and hemocytes in the metal rich cellular inclusions (Nunez-Nogueira et al., 2006). Zn is also found in insoluble inclusions along with P, Ca, Mg, and Si. The remainder of Zn is bound to the MTLP. Cadmium is predominantly present in the cytosolic fraction of the gill where it is detoxified by binding with MTLP.

In conclusion, two pools of MTLP exist in the branchial cytosol; one pool may be related to physiological regulatory processes and the other with transient changes in the free metal concentrations, depending on their fluctuations in the environment. Cu^2+^ and Zn^2+^ thioneins may be storage sites and potential donors for physiologically functional metalloproteins (i.e., CA and hemocyanin).

### Metal toxicity and gill physiology

Environmentally induced abiotic factors: temperature, salinity, pH changes; and biotic factors: moulting, sex, and reproductive cycle, may affect accumulation of toxic metals in aquatic organisms.

It is tempting to generalize that an increase in temperature would also result in an increase in metal toxicity. Toxic metals act on cellular enzymes involved in the production of metabolic energy, and an increase in environmental temperature would increase the uptake rate of metals and their toxic action in crustaceans. In the presence of toxic concentrations of copper, cadmium, and zinc, a small critical temperature increase of few degrees has a disproportionately large inhibition of oxygen consumption of the juvenile crayfish *Orconectes immunis* (Khan et al., 2006).

The rate of cadmium uptake by the gills of *Callinectes sapidus*, *Carcinus maenas*, and *Palaemonetes pugio*, increases in response to decreases in seawater salinity (Hutcheson, 1974; Wright, 1977; Engel and Fowler, 1979). One of the reasons for this is that the concentrations of free Ca^2+^ and Cd^2+^ are higher in lower salinities vs those in full strength seawater. Secondly, in hyperosmoregulating Crustacea, mechanisms of active ion transport are activated, and toxic metals compete with inorganic osmolytes for uptake by the gills.

Acidification of the aquatic environment lowers pH and reduces bioavailability of physiologically important free metals (e.g., Ca^2+^). Under these conditions, toxic metal ions, such as Cd, compete with the branchial carrier molecules and the efficiency of the transport processes decreases. This is believed to occur by proton (H^+^ ions) effects on Ca^2+^ channels which change the activity of Ca^2+^-activated Cl^−^ -channels (Barnes and Bui, 2003). The presence of such channels were suggested in lobsters epipodite (Towle and Smith, 2006; Lucu and Towle, 2010).

Biological factors, such as molting, also affect metal toxicity. Postmoult increase of cadmium in the shore crab, *Carcinus maenas*, may be a result of increased calcium transport and competition from Cd for the same transport mechanism (Norum et al., 2003). Copper distribution in the lobster *Homarus gammarus* and blue crab *Callinectes sapidus* during a moulting was shifted into the soft tissues (Hagerman, 1983; Engel, 1987), but total Cu^2+^ and Zn^2+^ content remain unchanged during a moult cycle. The changes in Cu^2+^ and Zn^2+^ concentrations that occurs in the late premoult stage were attributed to muscle breakdown and redistribution of metals, resorption from exoskeleton, and dilution of the hemolymph caused by entry of water. During a sheding of the shrimp, *Palemon elegans*, about 15% of Cd was lost, and shrimps took up additional Cd from the water (White and Rainbow, 1986).

Numerous biochemical and physiological functions are impaired by toxic metals. Copper exposure (100 μ g/L Cu^2+^) significantly reduced hemolymph Na^+^ and gill Na^+^/K^+^- ATPase activity in the freshwater amphipod *Gammarus pulex* (Brooks and Mills, 2003). Elevated concentration of ammonia in the hemolymph was caused by the increased concentration of copper and silver in the freshwater crayfish. Potential ammonia transport pathways might be linked to sodium transport, with both being affected by copper and silver exposure (Grosell et al., 2002).

High maximum capacity for Na^+^ influx stimulates copper and silver uptake in the freshwater crustacean, *Daphnia magna*, inhibiting Na^+^,K^+^- ATPase activity (Bianchini and Wood, 2003; Glover and Wood, 2005). The free Ag^+^ is most toxic to freshwater, then brackish, and then seawater animals. The toxicity is dependent upon its chemical form. Chloride ions can act in a protective role against Ag^+^ toxicity by the formation of AgCl_n_, which is less soluble and therefore a less toxic form of Ag (Wood et al., 1996).

Exposure of the Pacific white shrimp, *Litopenaeus vannamei*, to 3 mg/L Zn^2+^ or 3 mg/L Cd^2+^ for 24 h caused a 91 % and 76% inhibition of oxygen consumption, respectively, suggesting that the metals caused damage to the gills and disrupted the process of respiratory gas exchange (Wu and Cheng Chen, 2004). Long-term eposure to sublethal concentrations of zinc and cadmium induced impairment of respiratory functions under hyposmotic conditions in the crab, *Cancer pagurus*, by an increase in oxygen permeability of the gills and a decrease in the transfer factor of oxygen (Spicer and Weber, 1992).

The effect of lead toxicity on branchial cell volume regulation was studied comparatively in cells dissociated from the posterior gills of the osmoconforming marine *Hepatus pudibundus*, and the hyperosmoregulating estuarine *Callinectes ornatus*. In the gill cells incubated in isosmotic saline, cell volume decreased in response to 10 μM Pb^2+^ (Amado et al., 2012). This was explained by Pb^2+^ mimicking Ca^2+^ and thus activating Ca^2+^ sensitive K^+^ channels and/or Cl^−^ channels, resulting in outward K^+^ and Cl^−^ leakage and cell shrinkage (Wehner et al., 2003; Lang, 2007). When the Pb^2^ and Ca^2+^ channel inhibitor, verapamil, was added to the isosmotic saline, Ca^2+^/Pb^2+^ channels were blocked, and cell volume remained at the normal control values in both species. Isolated gill cells from the osmoconforming crab were still able to regulate volume under hyposmotic stress despite the addition of 10 μM Pb^2+^. However, under comparable conditions, isolated cells from the hyperosmoregulating crab could not adjust cell volume as well and were significanlty affected by the addition of Pb (Amado et al., 2012). In the presence of both the Ca^2+^ channel inhibitor verapamil and Pb^2+^, cell volume regulation was evident in both cell types. The authors explained the weaker volume regulation of the cells dissociated from hyperosmoregulating crustaceans under *in vitro* conditions not by the loss of the ability for extracellular anisosmotic regulation but as a result of increased capacity for anisosmotic extracellular regulation in hyper-regulators such as *Callinectes ornatus*. Extracellular regulation of osmolytes dampens changes in hemolymph osmotic concentrations and thus reduces need for (intracellular) cell volume changes (Foster et al., 2010).

Sublethal and chronic exposures of toxic metals induce numerous malformations and ultrastructural changes in the crustacean gill epithelium (Nonnotte et al., 1993; Victor, 1994; Lawson et al., 1995; Rtal et al., 1996; Powert et al., 1998; Hebel et al., 1999; Soegianto et al., 1999; Li et al., 2009; Issartel et al., 2010). Common malformations are characterized by thickened gill cells with reduced haemolymphatic space, cellular hyperplasia, and necrosis. Ultrastructural changes of the cells were demonstrated as a decrease of the plasma membrane infoldings, extensive increase of vacuole, malformations and decreased density of mitochondria, condensed nuclear matrix, and changed ribosomal distribution of the microtubular network.

### Models of trace metal transport across the gills

**Figure 10:**
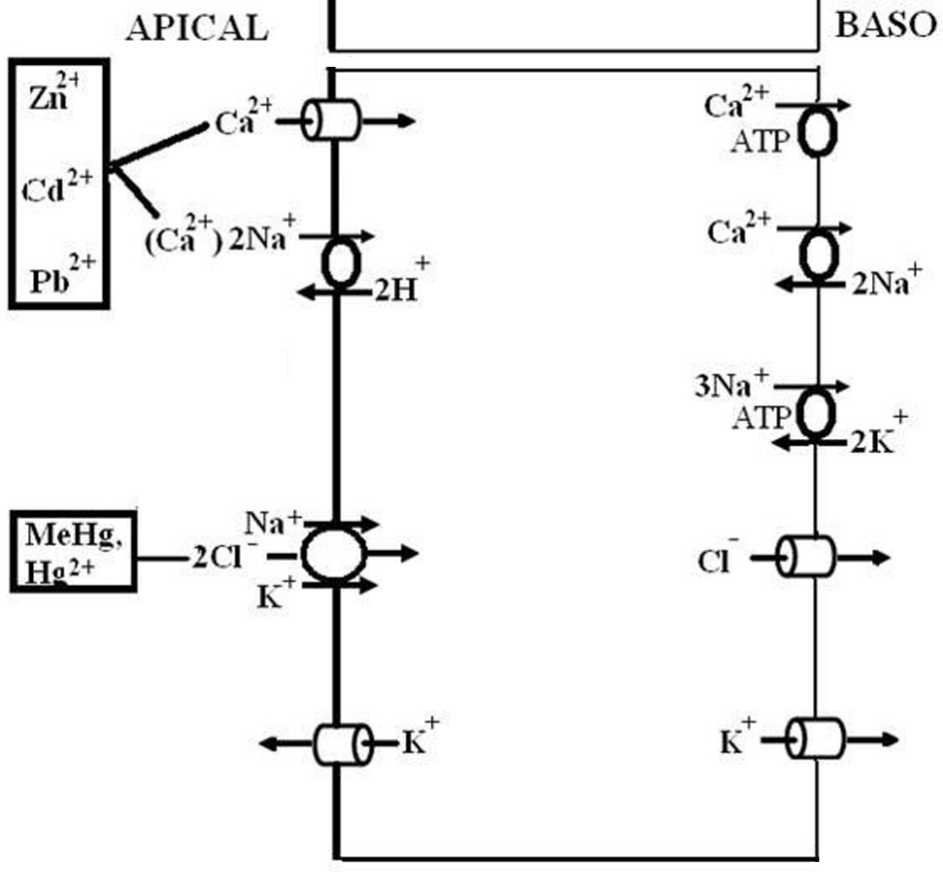
**Model of hypothetical transcellular uptake of the toxic divalent metals ions Zn^2+^, Cd^2+^, Pb^2+^, and MeHg across the gill epithelium of the weak and medium hypersomoregulating Crustacea is presented.** There are many evidences about apically located interactions of the divalent toxic metals with Ca^2+^ channel. An electroneutral Na^+^/H^+^ exchanger and Ca^2+^/2H^+^ as proposed by lobster hepatopancrease brush border membrane vesicles might be present. Non-specific inhibition of Na/K/2Cl cotransport by CH_3_HgCl and HgCl_2_ was reported. Basolaterally located Ca^2+^-ATPase and Ca^2+^/2Na^+^ exchange are candidates for basolateral exit of the trace metals. Numerous studies has shown inhibition of Na^+^,K^+^-ATPase by Cd^2+^, Pb^2+^, Zn^2+^, and consequently, secondary active transport mechanisms generated by this enzyme.

**Figure 11:**
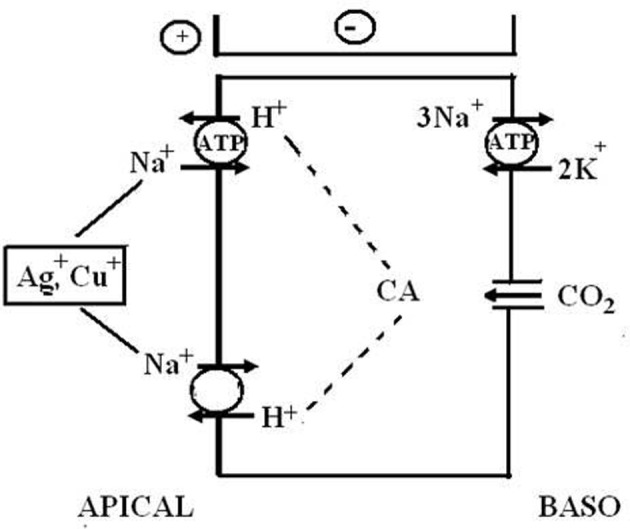
**Model of hypothetical transcellular pathway of the toxic metals Ag^+^ and Cu^+^ (after reduction of Cu^2+^ by cell surface enzymes reductase) across gill epithelium of strong hyperosmoregulating Crustacea (freshwater and brackish water Crustacea).** Toxic metals compete for putative Na-channel generated by V-type-H-ATPase, and/or electroneutral Na^+^/H^+^ exchanger or Ca^2+^/2H^+^ as proposed by lobster hepatopancrease brush border membrane vesicles. Toxic metals inhibits basolaterally located Na^+^, K^+^-ATPase, and carbonic anhydrase (CA). Later supplying counter ions H^+^ and HCO_3_ for ionic exchange by secondary generated transport mechanisms.

Hypothetical entry of toxic metals across the gill epithelium of marine and estuarine crustaceans is presented by a model based on ionic mimicry (Figures 10 and 11). Toxic metals interact with binding sites of membrane transport proteins and by this way are able to gain access to the intracellular compartments where they are a danger to normal biochemical and physiological functions (Ahearn et al., 2010; Wood, 2012).

Gills membrane transport mechanisms control the access of toxic metals and determine their uptake, distribution, and loss. Metals interact with apically located voltage-independent, non-selective Ca^2+^ channels (Figure 10). This model is based on experimental evidence indicating that Cd^2+^, Zn^2+^, and Pb^2+^ are transported across the crustacean gill epithelial cell by apically located voltage—independent Ca^2+^ channels driven by electrochemical gradients in Crustacea (Bjerregaard and Depledge, 1994; Pedersen and Bjerregaard, 2000; Ahearn et al., 2010) and fish (Wood, 2012). Toxic metals competes with the basolaterally located Na^+^/K^+^ -ATPase that generates apically and basolaterally located secondary active transport mechanisms. Also, a basolaterally located Ca^2+^ -ATPase (Morris and Greenaway, 1992; Wheatley and Gao, 2004) and/or Na^+^ /Ca^2+^ exchanger (Flik et al., 1994) are possible candidates for the transport of metals from the gill cells into the hemolymph. Uptake of Zn^2+^ and Pb^2+^ is blocked in the dissociated crustacean gill cells *in vitro* by Ca^2+^ channel inhibitors verapamil (Sa et al., 2009; Amado et al., 2012), and Cd^2+^ uptake is blocked by lanthanim (Lucu and Obersnel, 1996; Pedersen and Bjerregaard, 2000) and diltiazen (Borowitz and McLaughlin, 1992).

A second model, based on experimental results obtained on waterborne Ag^+^ and Cu^2+^ transport across the gills of freshwater and brackish water crustaceans, is also presented (Figure 11). Monovalent Ag^+^ competes with Na^+^ entry through putative apical Na^+^ channels; the movement of Na being generated by a V-type-H^+^-ATPase (neonate *Daphnia magna*) and/or Na^+^/H^+^ exchanger (adult *Daphnia magna*) (Bianchini and Wood, 2003; Glover and Wood, 2005). Cu^2+^ is imported into the cell in the reduced form Cu^+^through a Na^+^ channel (and/or Na^+^/H^+^ exchanger) in the freshwater amphipod, *Gammarus pulex* (Brooks and Mills, 2003; Martins et al., 2011).

In parallel with transport studies of toxic metals, it is important to also consider the chemical species of the metals in water and the cellular compartemnts in which they are sequestered as important information for understanding the transport pathways and ultimate fates of toxic metals that move across the gill epithelium. The fact that multiple toxic metals are emitted simulataneously into the environment requires studies on the evaluation of both synergistic and antagonistic effects during transport, including the potential for mutual interactions between metals. Studies on the specific transport mechanisms of essential metals (i.e., Cu^2+^- ATPase, Cu^2+^ transporters, and others) should be of importance for the better understanding of the transport of the toxic metals. The studies on the multifunctional role of the MT and the fate of intracellular and the intracellular granule and their further trafficking is also needed.

## Summary

The gills are the major site of interactions between waterborne toxic metals and the organism in aquatic Crustacea.

Experimental evidence for hyperosmoregulating brackish and freshwater Crustacea show that cationic species Cd^2+^, Zn^2+^, and Pb^2+^ can cross the gill epithelium at the apical side of the membrane by non-specific Ca^2+^ channels and other membrane transporters.

In the freshwater and brackish water Crustacea, Cu^2+^ and Ag^+^ compete with Na^+^ for uptake by the gill with the Na^+^/H^+^ exchanger, V-ATPase Cl^−^ exchangers, and Na^+^/K^+^/2Cl^−^ cotransporter.

Immunolocalization and biochemical techniques have shown that toxic metals inhibit the Na^+^/K^+^ -ATPase and CA, with implications for decreases in Na^+^ concentrations in hemolymph and consequent decreased osmoregulatory ability and damage of secondary active transport mechanisms.

The synthesis of metallothionein like proteins was induced by toxic metals (Zn^2+^, Hg^2+^, Cd^2+^), however, other factors such as hyposmotic salinity stress, temperature, and biotic factors (moulting, reproduction sex) may influence the activation of MT synthesis. Binding of toxic metals to MTLP and accumulation in intracellular vacuolar granules or extracellular granules is one of the mechanisms of metal detoxification.

### Conflict of interest statement

The authors declare that the research was conducted in the absence of any commercial or financial relationships that could be construed as a potential conflict of interest.
